# A Mathematical Model to Characterize the Role of Light Adaptation in Mammalian Circadian Clock

**DOI:** 10.3389/fmolb.2021.681696

**Published:** 2021-12-07

**Authors:** Yuzeng Shi, Yu Liu, Ling Yang, Jie Yan

**Affiliations:** School of Mathematical Sciences, Soochow University, Suzhou, China

**Keywords:** circadian clock, CRTC1-SIK1 pathway, light adaptation, phase robustness, singularity, refractoriness

## Abstract

In response to a light stimulus, the mammalian circadian clock first dramatically increases the expression of *Per1* mRNA, and then drops to a baseline even when light persists. This phenomenon is known as light adaptation, which has been experimentally proven to be related to the CRTC1-SIK1 pathway in suprachiasmatic nucleus (SCN). However, the role of this light adaptation in the circadian rhythm remains to be elucidated. To reveal the in-depth function of light adaptation and the underlying dynamics, we proposed a mathematical model for the CRTC1-SIK1 network and coupled it to a mammalian circadian model. The simulation result proved that the light adaptation is achieved by the self-inhibition of the CRTC1/CREB complex. Also, consistently with experimental observations, this adaptation mechanism can limit the phase response to short-term light stimulus, and it also restricts the rate of the phase shift in a jet lag protocol to avoid overly rapid re-entrainment. More importantly, this light adaptation is predicted to prevent the singularity behavior in the cell population, which represents the abolishment of circadian rhythmicity due to desynchronization of oscillating cells. Furthermore, it has been shown to provide refractoriness to successive stimuli with short gap. Therefore, we concluded that the light adaptation generated by the CRTC1-SIK1 pathway in the SCN provides a robust mechanism, allowing the circadian system to maintain homeostasis in the presence of light perturbations. These results not only give new insights into the dynamics of light adaptation from a computational perspective but also lead us to formulate hypotheses about the related physiological significance.

## Introduction

Circadian clock is an endogenous biological oscillator with a period close to 24 h in most organisms. Interlocked positive and negative transcriptional feedback loops are responsible for the self-sustained oscillations of circadian rhythms. In mammals, CLOCK/BMAL1 heterodimer is the master positive element in the circadian system which activates the transcriptions of *Per* and *Cry* genes through E-box. The negative elements of the oscillator are PER and CRY proteins which form PER/CRY heterodimers to inhibit their own transcriptions and other genes by inactivating the CLOCK/BMAL1 complex. Similar auto-regulatory negative feedback loops are also found in other organisms such as *Neurospora*, *Drosophila*, and *Cyanobacteria* (Ishiura et al., 1998; Glossop et al., 1999; Froehlich et al., 2003).

In addition to the self-sustained oscillation, the ability of entrainment to an exogenous cycle is also another important characteristic for the circadian rhythm to adapt to the environmental changes. Extensive research studies have already defined some external cues, such as light, temperature, and food availability, which can entrain the circadian clock (Freedman et al., 1999; Castillo et al., 2004; Refinetti, 2010). Light has been proven to be the strongest entraining factor for the cells in the suprachiasmatic nucleus (SCN). The molecular mechanism of light regulation on circadian genes has been explored in the past decades. In *Neurospora*, light acts on the circadian clock by promoting the transcription of *Frq* mRNA *via* the WCC (Crosthwaite et al., 1995; Lewis et al., 2002; He and Liu, 2005). The photic regulation in the circadian clock is also extended to mammals and *Drosophila*. In mammals, light induces the *Period* gene (*Per1* and *Per2*) *via* the cAMP pathway (Shigeyoshi et al., 1997; Yan et al., 1999; Field et al., 2000), while in *Drosophila* TIMELESS is rapidly degraded in response to light (Myers et al., 1996; Zeng et al., 1996).

However, further research revealed that abovementioned effect on the circadian clock by light is transient even if the organisms are exposed to prolonged light. This response to light is termed as light adaptation. The photoperiod tests performed in *Neurospora* showed that with the onset of the light phase, the level of *Frq* mRNA rapidly increases 10 times of the level under constant darkness in 30 min. Subsequently, the amount of *Frq* mRNA drops to less than 50% of the peak value and remains at this level for the rest of the light period (Tan et al., 2004). Molecular studies indicated that the negative regulation of the photoreceptor VIVID is responsible for this light adaptation (Heintzen et al., 2001; Gin et al., 2013). A similar phenomenon of light adaptation also exists in mammals. If the light pulse lasts for 6 h from CT17h, the level of *Per1* mRNA can increase sharply in 1 h; however, after that, the abundance of *Per1* mRNA gradually declines to the level before light stimulation (Vitaterna et al., 2006). This light adaptation enables the circadian clock to gain sensitivity to light changes, while attenuating the response to prolonged light exposure. Thus, it may be critical for organisms to adjust to the environmental changes appropriately.

Recent research found that the CRTC1-SIK1 pathway plays a key role in the entrainment of the circadian clock (Jagannath et al., 2013). In the SCN of mammals, light stimuli cause CRTC1 to co-activate CREB (cAMP response element binding protein), which upregulates the expressions of *Per* and *Sik1* by binding to cAMP response elements (CRE) in their promoters. In turn, SIK1 represses the activity of CRTC1 by phosphorylation. Therefore, a negative feedback loop between CRTC1 and SIK1 is confirmed. Naturally, two questions are raised: 1) How this negative feedback loop works in light adaptation? 2) What is the physiological function of light adaption in the mammalian circadian clock?

Mathematical modeling provides a powerful tool to understand the dynamics of genetic networks. Some mathematical models have been developed to explore the effect of light on circadian clocks (Kronauer et al., 1999; Locke et al., 2005; Tsumoto et al., 2011). Kunichika Tsumoto et al. used a 3-variable model to explore the effect of light adaptation on the entrainability of circadian rhythms by the light/dark cycles (Tsumoto et al., 2011). They found that the process of light adaptation can contribute to the adjustability of the circadian clock to 24-h light/dark cycles. Also, Richard E. Kronaue et al. developed a new model which includes a stimulus processor to predict the phase shifts to photic stimulus and the ability of entrainment (Kronauer et al., 1999). However, the molecular details involved in light effects were not incorporated into their models, and the function of light adaptation is still not fully understood.

Therefore, based on the regulatory circuits of the CRTC1-SIK1 pathway in mammals’ SCNs, we built a mathematical model and corroborated that the negative feedback loop in the CRTC1-SIK1 pathway generates light adaptation. Then, we coupled this module to a detailed mathematical model of the mammalian circadian clock which was developed by Mirsky et al. (2009). Using this model, we reproduced the observed experimental results (Jagannath et al., 2013), which showed that light adaptation can cause the circadian clock to avoid large phase variations upon short-term light stimulus and fast re-entrainment in advanced or delayed light/dark cycles. Therefore, light adaptation provides a phase robust mechanism for the circadian clock. The underlying reason of the mechanism was also investigated. Most importantly, we predicted that light adaptation can significantly reduce the possibility of singularity behavior, which implies the abolishment of the circadian rhythm by a critical light stimulus (Leloup and Goldbeter, 2001; Huang et al., 2006; Ukai et al., 2007). In addition, we also showed that light adaptation provides the refractoriness for the circadian clock. The results of this study suggest that the light adaptation generated by the CRTC1-SIK1 pathway favors the robustness of the circadian clock to resist deleterious environmental perturbations and maintain homeostasis.

## Results

### The Development of the Light-Dependent Model for the Mammalian Circadian Clock

**FIGURE 1:**
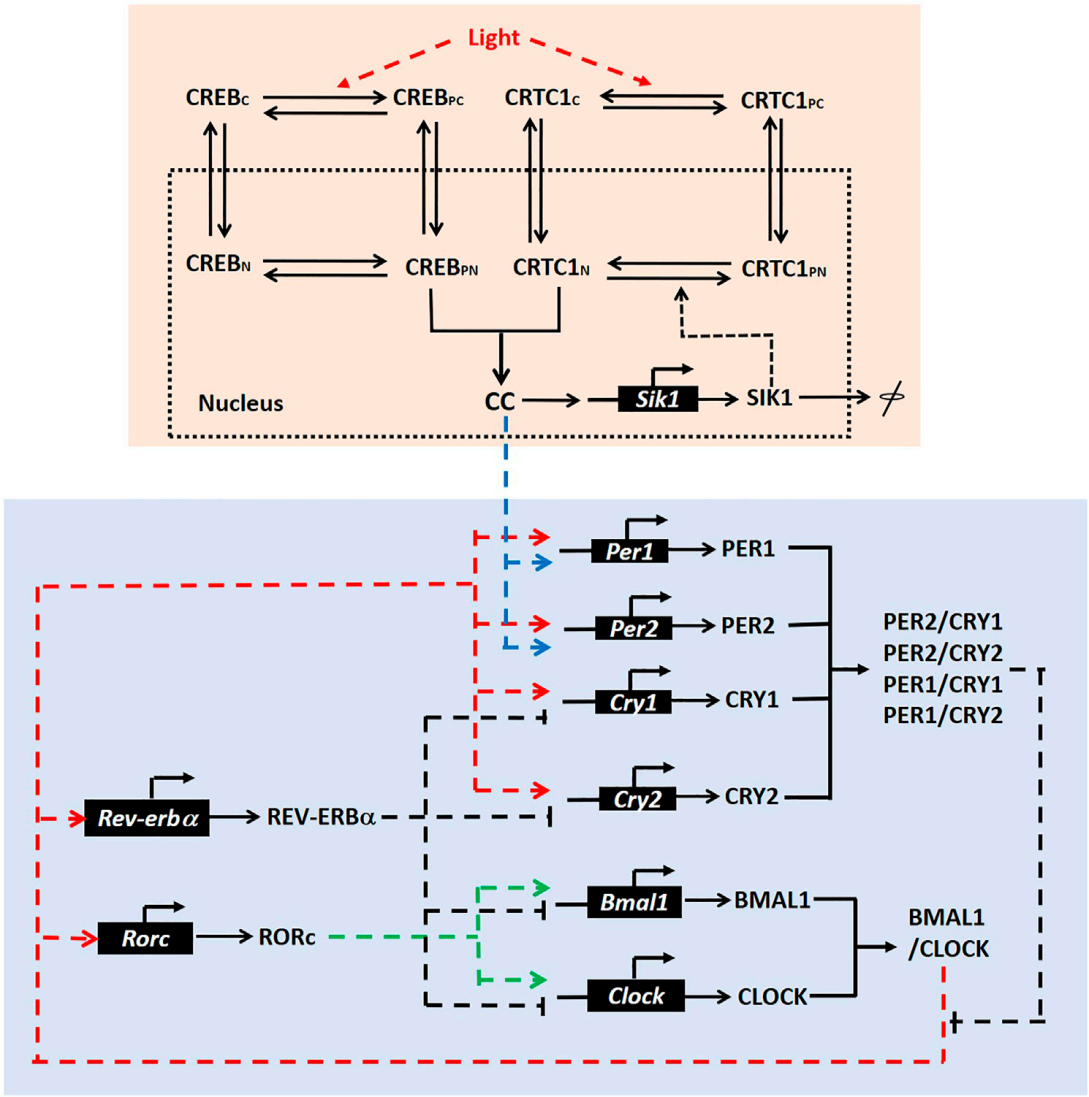
Model scheme. Pink box: The scheme for the CRTC1-SIK1 pathway in the SCN. We assumed four states for both CREB and CRTC1, which are denoted by CREB_C_ (cytoplasmic CREB), CREB_PC_ (phosphorylated CREB in cytoplasm), CREB_N_ (nuclear CREB), CREB_PN_ (phosphorylated CREB in nucleus), CRTC1_C_ (cytoplasmic CRTC1), CRTC1_PC_ (phosphorylated CRTC1 in cytoplasm), CRTC1_N_ (nuclear CRTC1), and CRTC1_PN_ (phosphorylated CREB in nucleus). CC represents the nuclear CREB/CRTC1 complex. Blue box: The regulatory circuit of the mammalian circadian clock, in which *Per1* and *Per2* are upregulated by the CRTC1-SIK1 pathway *via* the nuclear CREB/CRTC1 complex. This regulatory circuit is adapted from the schematic diagram of the circadian model in the reference (Mirsky et al., 2009).

To explore the effects of the CRTC1-SIK1 pathway on the mammalian circadian clock, we constructed a mathematical model for CRTC1-SIK1 regulatory circuits in the SCN and coupled it to a well-developed model of the mammalian circadian clock (Figure 1).

For the CRTC1-SIK1 module (Figure 1, pink box), light stimulus promotes the phosphorylation of CREB and the dephosphorylation of CRTC1 in the cytoplasm (Gau et al., 2002; Sakamoto et al., 2013). Phosphorylated CREB and dephosphorylated CRTC1 transfer into the nucleus and form the complex CREB/CRTC1, which is denoted by CC. Then, the complex CC activates the transcriptions of *Per1*, *Per2,* and *Sik1* by binding to cAMP response elements (CRE) in their promoters. Here, *Per1* and *Per2* are the two core circadian genes (blue box). Thus, light stimulus can induce the clock genes through the CREB/CRTC1 complex. SIK1, which is also promoted by CREB/CRTC1, can feedback to deregulate the CREB/CRTC1 complex by phosphorylating nuclear CRTC1. Therefore, the combination of CREB and CRTC1 upregulates the response of the circadian clock to light input, but SIK1 represses this response afterward. In this module, we simplified the posttranslational processes of SIK1 and only considered one form of the SIK1 protein. We also assumed that the total amounts of CRTC1 and CREB are constants, and thus omitted the synthesis and degradation of these proteins. This CRTC1/SIK1 model is governed by nine ordinary differential equations, which are listed in the Supplementary Material.

The core regulatory network of the mammalian circadian clock consists of a negative feedback loop and some auxiliary feedback loops. The CLOCK/BMAL1 complex induces the transcriptions of *Per* (*Per1*, *Per2*) and *Cry* (*Cry1*, *Cry2*). After that, PER and CRY form complexes and suppress their own transcriptions, which form the negative feedback loop (Figure 1, blue box). We coupled the CRTC1-SIK1 module to the 21-variable model of the mammalian circadian clock, which was developed by Mirsky et al. (2009). These two modules are connected by the induction of *Per1* and *Per2* transcriptions by the CREB/CRTC1 complex. Thus, we added a term dependent on the CREB/CRTC1 complex in the kinetic equations for the synthesis of *Per1* and *Per2* mRNA. The evolution equations of the CRTC1-SIK1 model and the circadian clock model, definitions, and values of parameters are given in the Supplementary Material (Supplementary Tables S1–S3).

### The Negative Feedback Loop in the CRTC1-SIK1 Pathway Generates Light Adaptation

**FIGURE 2:**
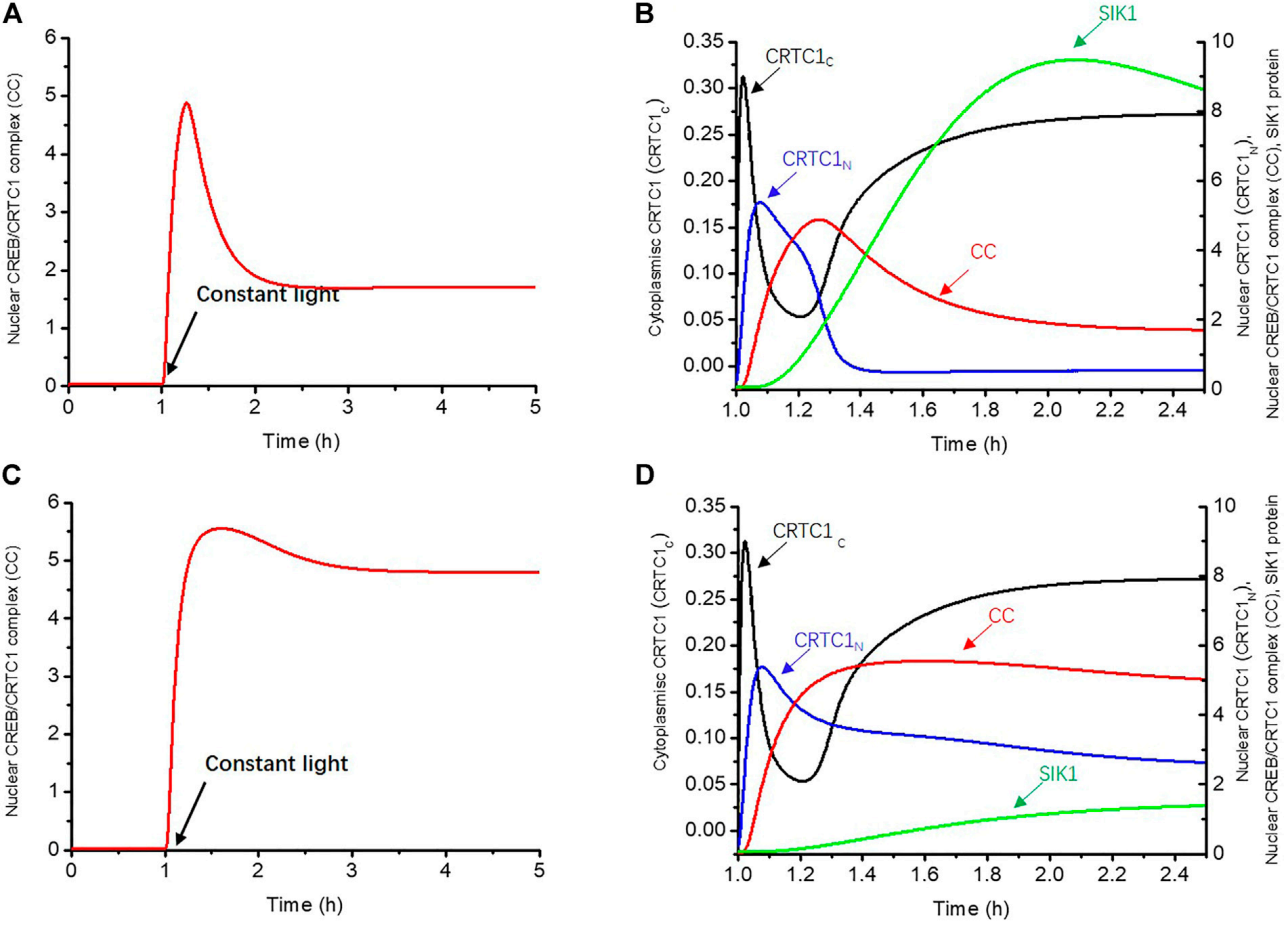
Negative feedback loop between the CREB/CRTC1 complex and SIK1 generates light adaptation. **(A)** In the WT, the nuclear CREB/CRTC1 complex rapidly increases as the light stimulus is applied, but drops to about one-third of its maximal level as light persists. **(B)** Time series of CRTC1-related proteins which are involved in transmitting the light effect to the CREB/CRTC1 complex in WT. Cytoplasmic CRTC1 (CRTC1_C_, black curve), nuclear CRTC1(CRTC1_N_, blue curve), and the CREB/CRTC1 complex (CC, red curve) are rapidly promoted by light in turn. However, with the accumulation of SIK1, a negative effect is triggered, resulting in the decline of the CREB/CRTC1 complex in the later period. **(C)** In *Sik1* knockdown mice, the CREB/CRTC1 complex increases quickly and only slightly drops upon light exposure. **(D)** Similar to WT mice, once the light stimulus is exerted, cytoplasmic CRTC1 (CRTC1c, black curve), nuclear CRTC1 (CRTC1n, blue curve), and the CREB/CRTC1 complex (CC, red curve) reach the maximal concentrations in order. However, the SIK1 protein (green curve) increases with slower speed due to the knockdown of *Sik1*. Thus, without sufficient SIK1 proteins, the CREB/CRTC1 complex is only slightly reduced afterward. The constant light stimulus starts at 1 h. The maximal synthesis rate of *Sik1* mRNA dependent on the CREB/CRTC1 complex *V1*_*sik1*_ is decreased from 20 to 2 to mimic the knockdown of *Sik1*. Parameter *L* increases from 0 to 40 to present the light stimulus. The initial conditions are listed in the Supplementary Material.

We first employed the isolated mathematical model for the CRTC1-SIK1 module to investigate the underlying mechanism of light adaptation. In the simulation, we used a parameter *L*, which multiplies the phosphorylation rate of CREB and the dephosphorylation rate of CRTC1 in the cytoplasm to mimic the promotion effects of light. The nuclear CREB/CRTC1 complex (CC) is considered an indicator of light-induced dynamics because light exerts its effects on circadian rhythms through the CC, which regulates the expressions of the circadian genes *Per1* and *Per2*. As shown in Figure 2A, we applied the constant light stimulus at 1 h, and the time course of the CREB/CRTC1 complex exhibits light adaptation under constant light (Figure 2A). The level of the CREB/CRTC1 complex first quickly increases at the beginning of the exposure to light and peaks at 1.27 h, which shows the sensitivity of the CRTC1-SIK1 module to the light stimulus. Over time, the CREB/CRTC1 complex begins to decline and eventually stabilizes at about one-third of the maximal level, exhibiting the attenuated response to prolonged light exposure.

To further reveal how this light adaptation occurs, we then showed the time series of dephosphorylated CRTC1-related variables (CRTC1_C_, CRTC1_N_) which are involved in transmitting the light effect to the CREB/CRTC1 complex (Figure 2B). Once the light stimulus is given at 1 h, dephosphorylated CRTC1 in the cytoplasm increases rapidly due to light promotion and reaches the maximal concentration at 1.02 h (black curve, CRTC1_C_). Subsequently, these surging CRTC1s are transferred into the nucleus, leading to a delayed peak of nuclear CRTC1 at 1.08 h (blue curve, CRTC1_N_). The increase of dephosphorylated nuclear CRTC1 is followed by the accumulation of the CREB/CRTC1 complex (red curve, CC), which peaks at 1.27 h. Therefore, this positive feedforward regulation from light to the CREB/CRTC1 complex can enable the circadian clock to respond rapidly to light stimulus. However, during the increase of the CREB/CRTC1 complex, the protein SIK1 also gradually rises (green curve) due to positive regulation of the CREB/CRTC1 complex. It is worth noting that the increase of SIK1 (green curve) is accompanied with the decrease of dephosphorylated CRTC1 in the nucleus (blue curve, CRTC1_N_,), as a result of SIK1-promoted phosphorylation. When SIK1 further accumulates, its negative regulation begins to take stronger effect, leading to the decline of the CREB/CRTC1 complex (red curve, CC). Thus, the negative feedback loop between the CREB/CRTC1 complex and SIK1 results in attenuated response to the prolonged light stimulus, and the CRTC1-SIK1 module is responsible for the generation of light adaptation.

We then disrupted this negative feedback loop by knocking down *Sik1* mRNA in the mathematical model to further verify that the light adaptation is due to the negative feedback between SIK1 and the CREB/CRTC1 complex (Figures 2C,D). The constant light stimulus is applied at 1 h. Since the positive forward regulation from light to the CREB/CRTC1 complex is intact, the concentrations of dephosphorylated CRTC1 in the cytoplasm (black curve) and nucleus (blue curve), as well as the CREB/CRTC1 complex (red curve), rapidly increase in turn at the beginning of the light phase (Figure 2D). However, light adaptation does not occur since the CREB/CRTC1 complex remains at a high level as light persists. This is reasonable because the SIK1 protein cannot effectively inhibit the formation of the CREB/CRTC1 complex. As a consequence, the level of the CREB/CRTC1 complex is only slightly decreased because of the residual SIK1 protein (Figure 2D).

Comparison of the light responses in WT (wild-type) and *Sik1* knockdown models supports the conclusion that the CRTC1-SIK1 module is the underlying mechanism of light adaptation; the positive forward regulation from light to the CREB/CRTC1 complex enables the circadian clock to gain sensitivity to light variation, while the negative feedback loop between the CREB/CRTC1 complex and SIK1 prevents further induction by light.

To test the generality of the conclusion, we randomly generated 10,000 parameter sets for the CRTC1-SIK1 module (Supplementary Table S4.). For each parameter set, we evaluated the magnitude of light adaptation by the ratio of the stabilized level of CC to the maximal value in the light phase,

$$\text{The magnitude of light adaptation}=\frac{\text{the stabilized level of CC}}{\text{ the maximal value of CC}}.$$ (1)

Therefore, a smaller ratio implies stronger light adaptation. The simulation results showed that 3,728 out of 10,000 parameter sets yield ratios smaller than 0.8, which indicates the occurrence of light adaptation. By these parameters, if *Sik1* mRNA is knocked down, the CRTC1-SIK1 module exhibits weakened light adaptation for 3,149 parameter sets (Supplementary Figure S1). Among these parameters, the magnitudes of light adaptation are weakened more than 20% for 2,882 parameter sets. Therefore, this result further confirms the conclusion that the negative feedback loop of SIK1 is essential for the generation of sufficient light adaptation.

### Light Adaptation Enhances the Phase Robustness by Limiting Phase Variations of the Circadian Clock

Sudden and dramatic changes usually happen in the external environment, such as light, food, and activity. Directly transmitting these changes to the circadian network may disrupt the balance between the physiological systems throughout the body. Limiting the effects of external stimuli is necessary to prevent such disruptions. Previous experimental evidences indicated that the CRTC1-SIK1 pathway can restrict the phase change of the circadian clock (Jagannath et al., 2013). *Sik1* knockdown mice exhibit larger phase-shifting response to short-term light than WT mice. Also, *Sik1-*silenced mice are more likely to be overwhelmed by varied photoperiodic cycles, showing speedy re-entrainment to the experimental jet lag protocol. One possible explanation for this phenomenon could be that the oscillation of the circadian clock in *Sik1* knockdown mice is weakened, leading to more sensitivity to light stimuli and faster re-entrainment. However, experimental data showed that the amplitude of *Per1* mRNA is only slightly changed in *Sik1* mutant mice(Jagannath et al., 2013). Therefore, some other mechanisms may contribute to the phase regulation. In this section, we incorporated the CRTC1-SIK1 module into the circadian clock and explained how the light adaptation module limits the phase-shifting effect in the circadian clock to enhance the phase robustness.

**FIGURE 3:**
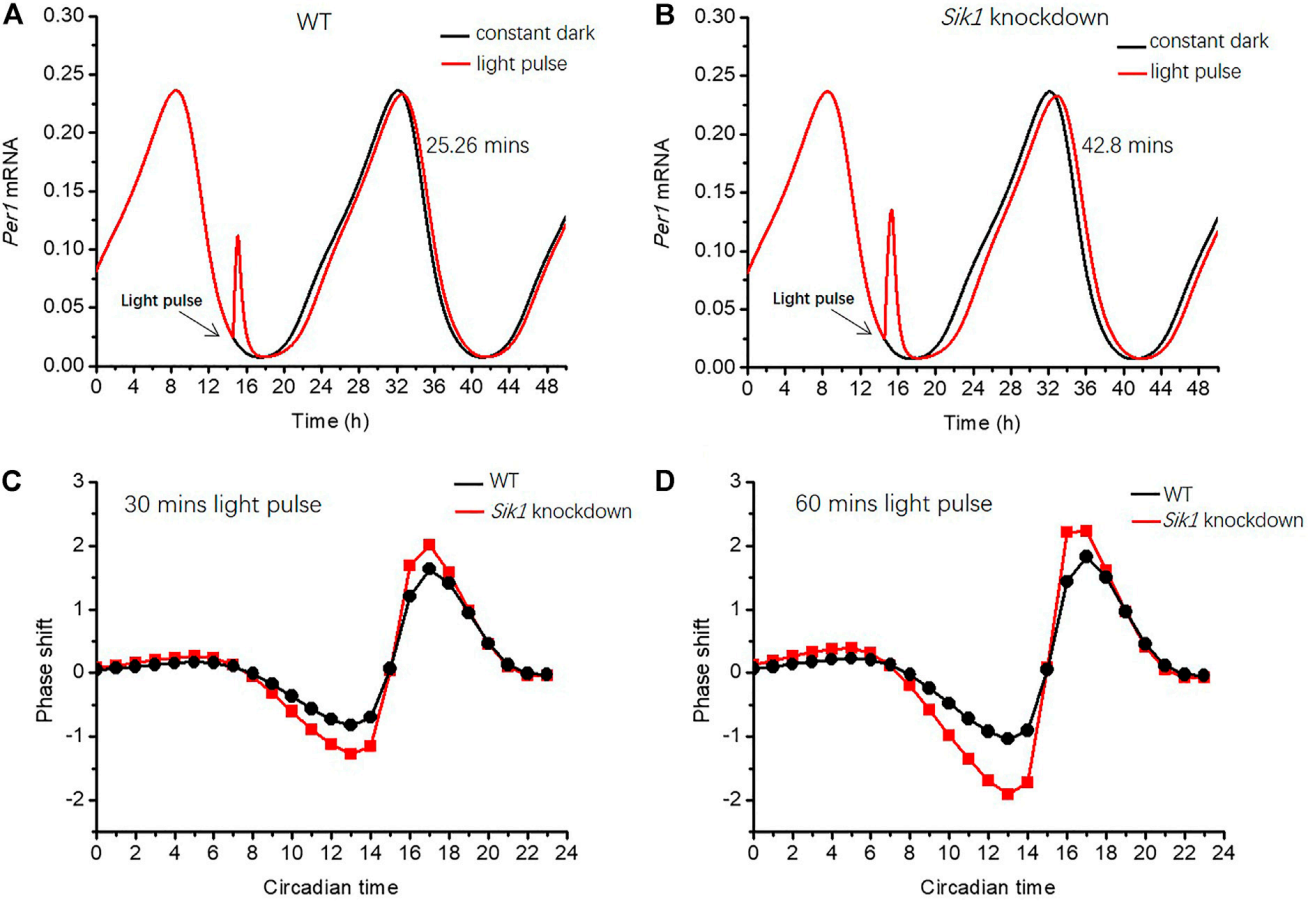
Light adaptation limits the phase-shifting effect of the circadian rhythm **(A–B)** A light pulse with a duration of 30 min is given at CT14.5 h (as indicated by the black arrow) in both WT and *Sik1* knockdown mice. The phase shift is defined by the time interval between the first peaks of *Per1* mRNA after the light pulse (red curve) and without light pulse (black curve). The phase of *Per1* mRNA is delayed by 25.26 min in WT **(A)**, while the delay increases to 42.8 min in *Sik1* knockdown mice **(B)**. Two different light pulses with a duration of 30 min and 1 h from CT0h to CT24 h (with an interval of 1 hour) are given, and the related phase response curves are exhibited in **(C)** and **(D)**. In both situations, the magnitude of the phase shift is larger in *Sik1* knockdown mice (red) than that in WT (black). Parameter *L* increases from 0 to 40 to present the effect of the light stimulus. *V1*_*sik1*_ is decreased from 20 to 2 to mimic the knockdown of *Sik1*. CT0h is determined by assuming that *Per1* mRNA peaks at CT8.5 h according to previous experimental results.

We first exhibited the effect of a short-term light pulse on the circadian phase. In our simulations, *Per1* mRNA is selected to indicate the phase shift of the circadian clock. We increased the parameter of light effect *L* from 0 to 40 at CT14.5 h for 30 min to mimic the light pulse. CT0h is determined by assuming that *Per1* mRNA peaks at CT8.5 h according to previous experimental results (Ueda et al., 2005). The expressions of *Per1* mRNA in the WT and *Sik1* knockdown models are depicted in Figures 3A,B. The phase shift is calculated by the time interval between the first peaks of *Per1* mRNA after the light pulse (red curve) and without the light pulse (black curve). The simulation results showed that the phase-shifting in the WT and *Sik1* knockdown models is 25.26 min (Figure 3A) and 42.8 min (Figure 3B) respectively. This result fits well with the experimental observation that the phase-shifting in *Sik1* knockdown mice is significantly increased after a short light stimulus.

To gain global insights into the phase shifts in WT and *Sik1* knockdown mice, we extended the results by exerting the light pulse at different times and drew the related phase response curves (PRCs). The PRCs were simulated for two different light stimuli, with a duration of 30 min (Figure 3C) and 1 h (Figure 3D), respectively. As shown in Figures 3C,D, the phase shifts in *Sik1* knockdown mice are obviously enhanced in both cases. Even if the phase of *Per1* mRNA is delayed or advanced, the magnitude of the phase shift is larger in *Sik1* knockdown mice. Therefore, the module of light adaptation prevents the circadian system from the perturbations of transient light pulse by restricting the phase-shifting effects.

Other parameter sets were also tested by applying a 30-min light pulse at about CT14.5 h (Supplementary Figure S2). We excluded the parameter sets that destroy the self-oscillation of the circadian clock from 2,882 parameter sets, for which the magnitudes of light adaptation are weakened more than 20% in *Sik1* knockdown mice. Thus, 2,287 parameter sets were obtained to check the phase shift response to short-term light pulse. As the circadian clock with the CRTC1-SIK1 module exhibits stable oscillation, the 30-min light pulse is applied at 6 h after the peak of *Per1* mRNA. For most parameter sets, the phases of *Per1* mRNA are delayed. By measuring the phase shifts (the time interval between the first peaks of *Per1* mRNA after the light pulse and without light pulse) in the WT and *Sik1* knockdown models, we can observe that the mutant model exhibits larger phase shifts for 2,285 out of 2,287 parameter sets (Supplementary Figure S2A). Furthermore, the phase shift is increased by 20% in the *Sik1* knockdown model for 1,680 parameter sets. We also found that for some parameter sets, when the amplitude of the circadian oscillation is small, the phase shifts of the WT and *Sik1* knockdown models are much larger. Supplementary Figures S2B–C present an example for this situation, where the difference of phase shifts between the WT and *Sik1* knockdown models is −3.38 h. Therefore, the conclusion that the light adaptation generated by the CRTC1-SIK1 module preventing excessive phase shift still holds true for most parameter sets.

**FIGURE 4:**
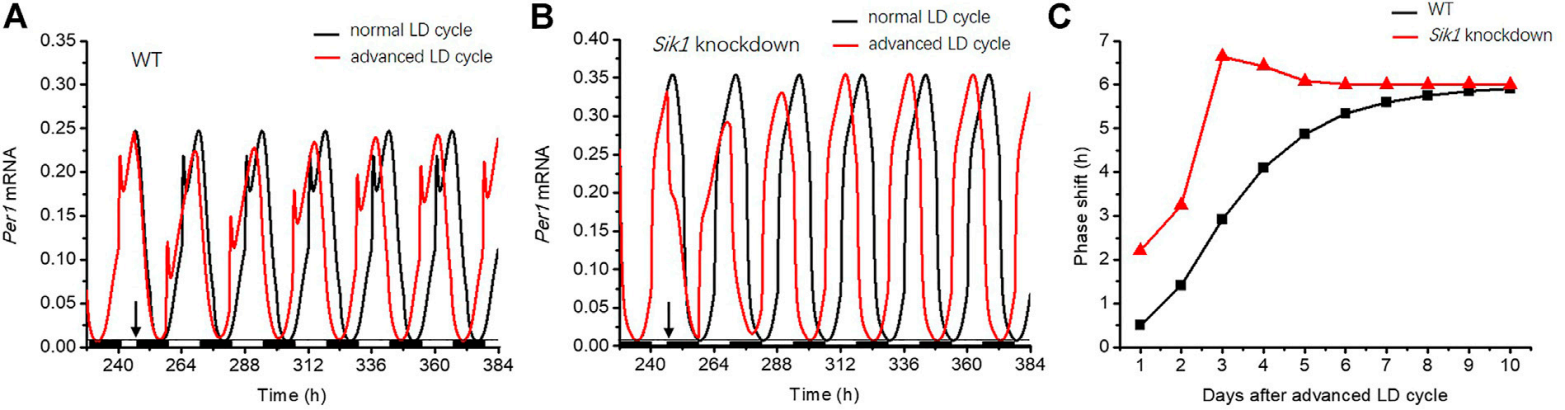
Light adaptation limits the rate of re-entrainment upon the advanced LD cycle. The circadian clock is entrained by a 12:12 LD cycle for 10 days, and the dark phase is advanced by 6 h from the 11^th^ cycle (as indicated by the black arrows). The time series of *Per1* mRNA in WT **(A)** and *Sik1* knockdown mice **(B)** are exhibited. The black and red curves represent the time evolution of *Per1* mRNA without the advanced LD cycle and after the advanced LD cycle. The black and white bars on the bottom of the horizontal axis represent dark and light phases, respectively. **(C)** Daily changes of the phase in response to the advanced LD cycle are measured, which show larger daily variations in *Sik1* knockdown mice (red) than that in WT (black). *Sik1* knockdown mice can be entrained to the new LD cycle more quickly. The values of the parameters are the same as those in Figure 3.

Then, we performed the simulations of the circadian clock following the jet lag protocol as described in the reference (Jagannath et al., 2013), to explore the role of light adaptation in re-entrainment. The circadian clock is entrained by the 12:12 LD cycles for 10 days, and then the dark phase is advanced by 6 h in the 11^th^ cycle. The related time series of *Per1* mRNA in the WT and *Sik1* knockdown models are shown in Figures 4A,B. The red curve represents the time evolution of *Per1* mRNA upon the advanced LD cycle, which is applied at 246 h as the black arrow indicates. To observe how the phase changes, we also exhibited *Per1* mRNA without the advance of the LD cycle (Figures 4A,B, black curve). As shown in Figures 4A,B, the phase variation in *Sik1* knockdown mice is larger when the LD cycle is advanced. We then quantified the daily phase variation by measuring the time intervals between the large peaks of the red curve and the black curve of each day (Figure 4C). It is clear that the phase variation in WT case is more moderate (black, Figure 4C). The phase is gradually re-entrained to the advanced LD cycle. However, in *Sik1* knockdown mice, the re-entrainment process is faster, and the phase variation at the beginning of the varied photoperiodic cycle is much larger than that in WT mice. These simulation results agree well with the experimental observation that knockdown of *Sik1* causes more significant behavioral phase shifts and rapid re-entrainment (Jagannath et al., 2013). Moreover, we extended the results to other scenarios when the LD cycle was advanced by 12 h, delayed by 6 h, and again delayed by 12 h (Supplementary Figure S3). Similar to what is shown in Figures 4A,B, the *Sik1* knockdown model exhibits faster re-entrainment than the WT model in these three cases.

**FIGURE 5:**
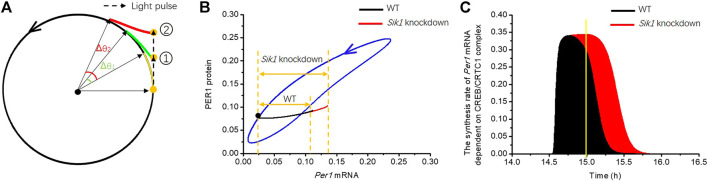
Dynamical mechanism underlying the regulation of phase-shifting **(A)** Phase plane of a conceptual model displaying limit cycle behavior is exhibited, which has a strongly attractive circular orbit (black circle) around an unstable fixed point (black dot). A light pulse (black dashed arrow) is applied as the orbit runs to the yellow point on the circle, and the orbit is pushed to position 1 after the stimulus (dashed arrow). As the orbit returns to the limit cycle (green curve), its phase is shifted by Δθ_1_ compared with the orbit without the stimulus (yellow curve). If the stimulus is stronger, the orbit will be pushed to position 2 , so that a larger phase shift Δθ_2_ occurs (red curve). The black solid arrows indicate the shift phase. **(B)** In the phase plane of *Per1* mRNA—PER1 protein, the blue closed curve is the limit cycle of the circadian clock in constant darkness. The orbits, changed by the 30-min light pulse at CT14.5 h (black dot) in WT and knockdown mice, are shown by the black and red curves, respectively. *Per1* mRNA in the *Sik1* knockdown mice exhibits significantly larger traveling distance (the double arrows) upon light stimulus. **(C)** Time series of the synthesis rate of *Per1* mRNA dependent on the CREB/CRTC1 complex indicates the underlying dynamics. The filled areas represent the accumulation of *Per1* mRNA dependent on light stimulus in WT (black) and *Sik1* knockdown (red) mice, which determine the traveling distance of *Per1* mRNA in the phase plane. The yellow line indicates the time when the light pulse ends.

The model was further used to investigate how light adaptation regulates the phase of the circadian clock. Before considering the detailed dynamics of this system, it is useful to resort to a conceptual model displaying limit cycle behavior to present the basic idea. The topological structure of the circadian oscillator in constant darkness is similar to that of a dynamical system with a limit cycle (Ukai et al., 2007). A model with a limit cycle has a strongly attractive circular orbit around an unstable fixed point (Figure 5A); therefore, if an orbit starts from the limit cycle, it keeps running on it. Otherwise, it will be attracted to the cycle. Figure 5A shows that if a light stimulus is applied when the orbit arrives at the yellow point, the orbit is forced to leave the circular limit cycle. Then, as soon as the light pulse is removed, the orbit tends to return to the limit cycle from position 1 (green curve). It is worth noting that, compared with the original oscillator without the light pulse (yellow curve), the phase of the stimulated oscillator has been shifted by Δθ_1_ when it returns back to the limit cycle (green curve). If the orbit is driven further from the limit cycle by a stronger light stimulus to position 2 , a bigger phase shift Δθ_2_ will occur (red curve).

Let us see how the scheme of Figure 5A helps us understand the underlying mechanism of limiting phase-shifting by light adaptation in the detailed model. We used the light pulse of 30 min at CT14.5 h as an example to analyze the phase response of the circadian clock. The phase shift is enhanced (25.26 mins vs. 42.8 min) in *Sik1* knockdown mice, as shown in Figures 3A,B. We exhibited the related phase plane of *Per1* mRNA and PER1 protein in Figure 5B. The blue closed curve is the limit cycle of WT and *Sik1* knockdown mice in constant darkness. The black dot on the cycle corresponds to CT14.5 h. As the 30-min light pulse is applied, it pushes *Per1* mRNA out of the limit cycle in both WT (blue curve) and *Sik1* knockdown mice (red curve). Moreover, as indicated by the double arrows, the movement of *Per1* mRNA in *Sik1* knockdown mice goes further away from the limit cycle than that in WT mice. The distance of *Per1* mRNA between the light-pushed orbit and the limit cycle is determined by mRNA accumulation on stimulus. To estimate the light-induced mRNA accumulation, we may go back to the ordinary differential equation governing *Per1* mRNA, which is listed in the Supplementary Material.

$$\frac{dmPer1}{dt}=\left(V0_{P1}+V1_{P1}\frac{CLK\_BMAL1^{na1\_P1}}{KA1_{P1}^{na1\_P1}+CLK\_BMAL1^{na1\_P1}}\right)\cdot \frac{KI1_{P1}1^{ni1\_P1}}{KI1_{P1}^{ni1\_P1}+PER1\_CRY1^{ni1\_P1}}\cdot \frac{KI2_{P1}^{ni2\_P1}}{KI2_{P1}^{ni2\_P1}+PER1\_CRY2^{ni2\_P1}}\cdot \frac{KI3_{p1}^{ni3\_P1}}{KI3_{p1}^{ni3\_P1}+PER2\_CRY1^{ni3\_P1}}\cdot \frac{KI4_{P1}^{ni4\_P1}}{KI4_{P1}^{ni4\_P1}+PER2\_CRY2^{ni4\_P1}}+V2_{P1}\cdot \frac{CC^{m2\_CC}}{K2_{CC}^{m2\_CC}+CC^{m2\_CC}}-km_{P1}\cdot mPer1\text{.}$$

The accumulation of light-induced *Per1* mRNA originates from the nuclear CREB/CRTC1 complex (CC). Thus, we exhibited the time series of the synthesis rate of *Per1* mRNA dependent on the nuclear CREB/CRTC1 complex ( $V2_{P1}\frac{CC^{m2\_CC}}{K2_{CC}^{m2\_CC}+CC^{m2\_CC}}$ ) (Figure 5C). The filled area under the curve represents the amount of *Per1* mRNA accumulation induced by the CREB/CTRC1 complex. Clearly, the amount of light-induced *Per1* mRNA in *Sik1* knockdown mice (red zone) is much larger than that in WT mice (black zone). This difference in area is mainly caused by the SIK1-related adaptation. In WT mice, the synthesis rate dependent on CC increases rapidly at the beginning of the light pulse, leading to triggering of the negative feedback regulation by SIK1. Therefore, the rate has already declined before the end of the light pulse (as indicated by the yellow line). By contrast, in *Sik1* knockdown mice, the transmitting pathway from the light pulse to CC still works, so that the synthesis rate of *Per1* mRNA dependent on CC reaches the maximal level soon. However, due to lack of sufficient SIK1 proteins, the synthesis rate remains at the high level even when the light is removed. Consequently, the amount of light-induced *Per1* mRNA is significantly larger in *Sik1* knockdown mice. As a result, the light-pushed distance of *Per1* mRNA from the limit cycle on the phase plane is further (Figure 5B), leading to a larger phase shift.

Analyzing the response of *Per1* mRNA to light stimulus helps us understand the mechanism by which light adaptation limits the phase variation: In the WT mice, the light adaptation provided by the CRTC1-SIK1 pathway suppresses the response to the light pulse. This reduces the mRNA traveling distance away from the limit cycle and thereby maintains the phase shift in a suitable range. Such a limitation of the phase shift to a light pulse can be viewed as a buffering mechanism of enhancing the robustness of the circadian phase. For the jet lag experiments, adaptation provided by SIK1 prevents excessive phase shift day by day. Therefore, the WT circadian clock usually takes more days to adjust to new LD cycles.

In summary, the light adaptation limits the phase-shifting effect by reducing the mRNA traveling distance in phase space, thereby preserving the phase change in a modest range.

### Light Adaptation May Prevent the Occurrence of Singularity Behavior

Singularity behavior represents the worst situation in the circadian clock. It means that robust circadian rhythms can be abolished by a certain stimulus in cell population, such as the pulse of light at an appropriate time with right strength (Huang et al., 2006). The experimental data showed that desynchronization of an individual cellular clock underlies singularity behavior (Ukai et al., 2007). Because many physiological processes rely on a robust circadian clock, the organisms should have a mechanism to avoid or reduce singularity behavior.

**FIGURE 6:**
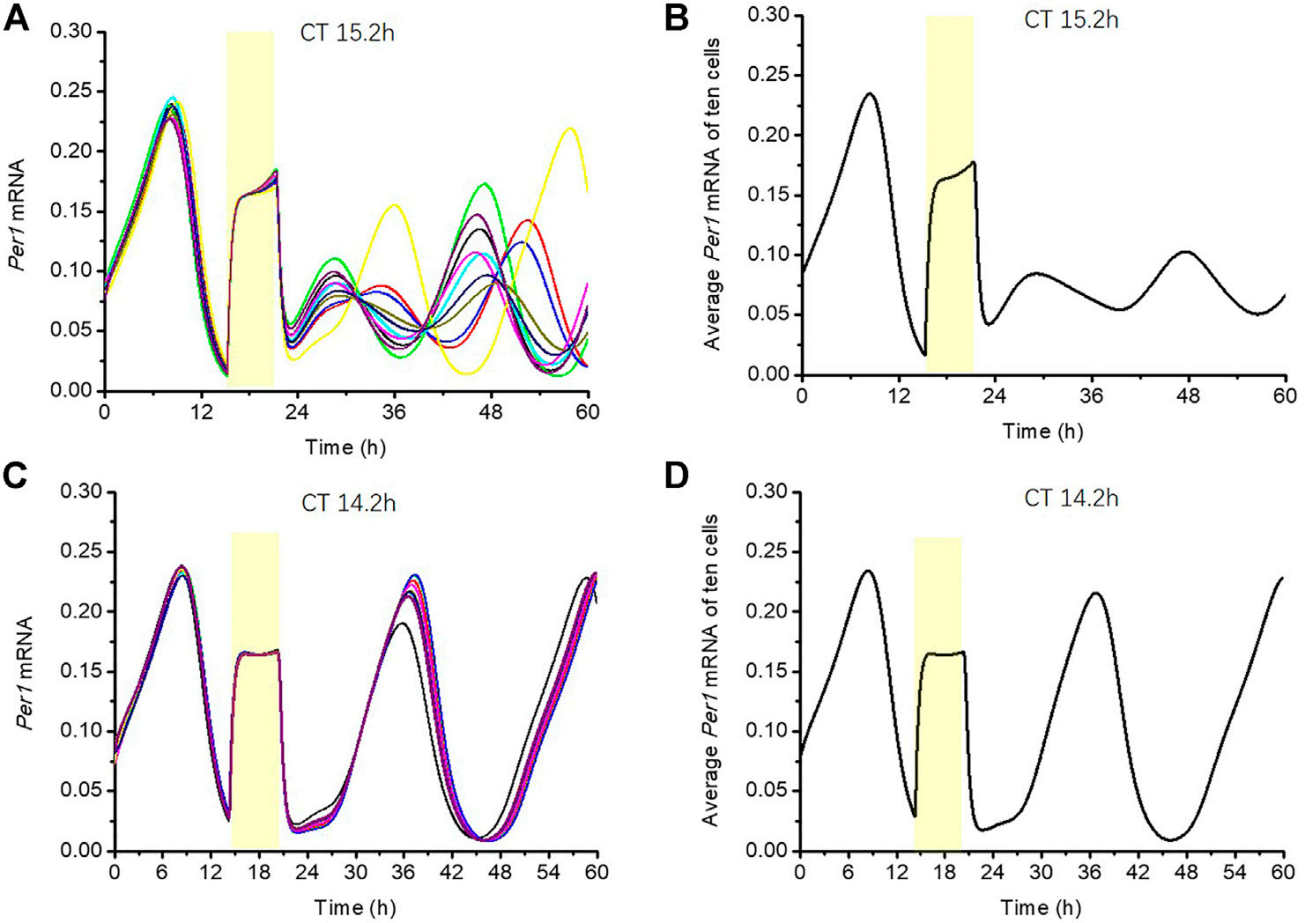
Phenomenon of singularity behavior in *Sik1* knockdown mice **(A)** 10 cells with slightly different initial conditions (phases) are stimulated by a 6-h light pulse at CT15.2 h (as indicated by the yellow zone). The time series of *Per1* mRNA in these ten cells display wide phase distribution after the light stimulus. **(B)** Average amplitude of *Per1* mRNA of the ten cells shows weak rhythmicity. The weakening of the average output amplitude reflects the fact that the light pulse brings the cells in the vicinity of the singularity, that is, the unstable steady state around which the oscillations develop. **(C)** If the light is given at CT14.2 h for 6 h (as indicated by the yellow zone), these ten cells are all nearly synchronized **(D)** Average amplitude of *Per1* mRNA almost remains unchanged, reflecting the fact that the light pulse does not bring the cells close to the singularity point. The initial conditions of the ten cells are randomly distributed in a small neighborhood (+/−5% of the amplitude) of a point on the stable periodic orbit before the stimulus (listed in the Supplementary Material).

In this section, we used the circadian clock model with the CRTC1-SIK1 module to illustrate the singularity behavior under a certain stimulus for the *Sik1* knockdown model (Figure 6). Ten single cells run along the stable periodic orbit with slightly different phases. Figure 6A shows the response of the ten cells to a stimulus at CT15.2 h with a duration of 6 h in *Sik1* knockdown mice. These cells are completely desynchronized after the stimulus, and the average *Per1* mRNA basically loses the rhythmicity (Figure 6B). This abolishment of the circadian rhythm, which is due to desynchronization, is called singularity behavior. However, Figure 6C displays a totally different pattern when the light pulse is given at CT14.2 h. Ten cells are yet almost synchronized after the stimulus, and the average output is rhythmic, similar to the single cell (Figure 6D). The simulation confirms that the singularity behavior can be induced by a light pulse at an appropriate time with the right magnitude, and desynchronization of an individual cellular clock is a key feature for circadian rhythm suppression at a global level.

**FIGURE 7:**
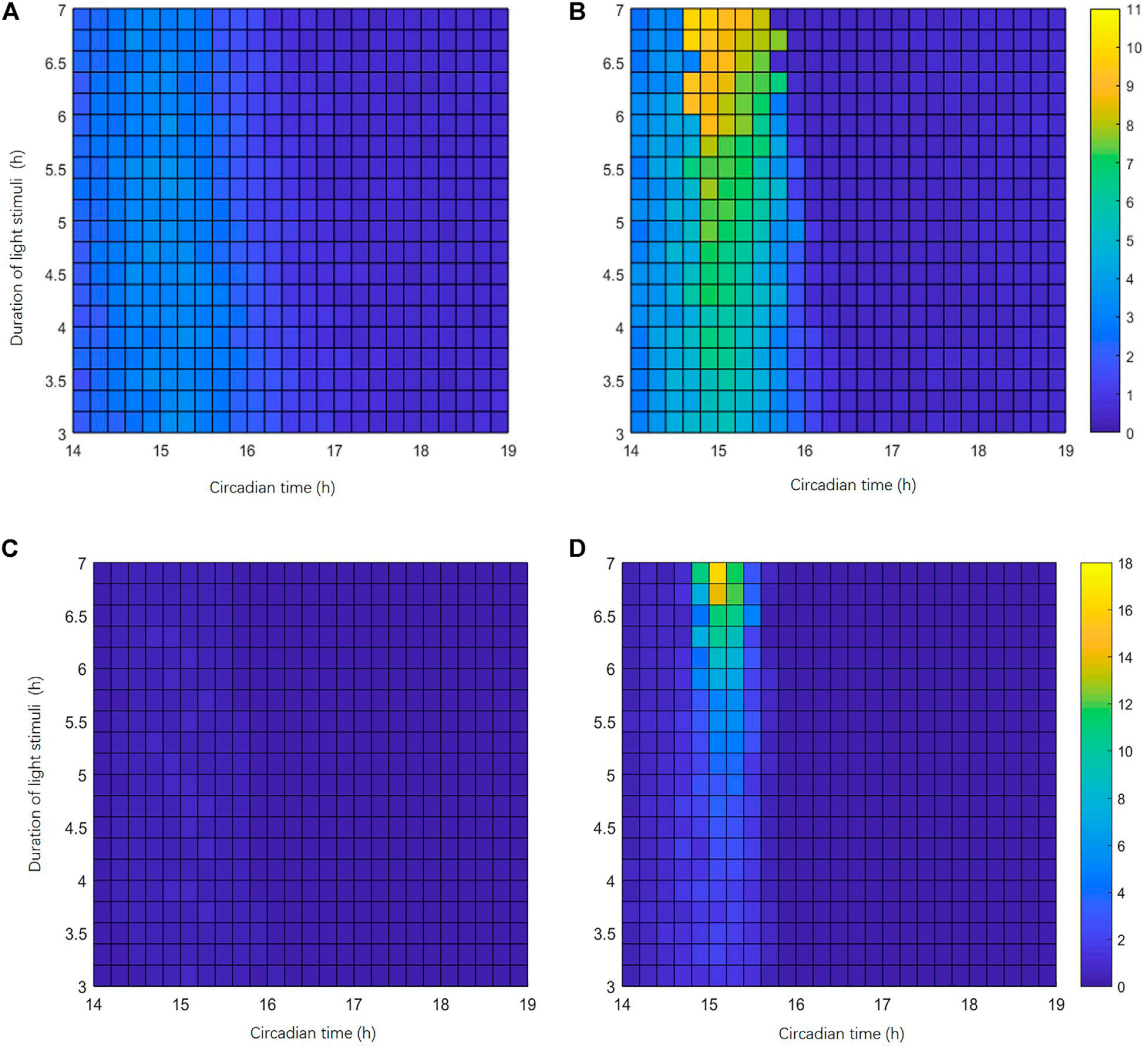
Singularity behavior is more likely to happen in *Sik1* knockdown conditions. **(A)** Heatmap representation of the largest phase difference (MPD) of the circadian clock in 100 WT cells (24 h after the light stimulation). The initial conditions of these 100 cells are randomly distributed in a small neighborhood (+/−5% of the amplitude) of a point on the stable periodic orbit before stimulus. The stimuli of variable magnitudes are given at different phases occurring from CT14 h to CT19 h (interval of 12 min, *x*-axis), with light pulse durations ranging from 3 h to 7 h (interval of 12 min, *y*-axis). The heatmap thus includes 26 × 21 points. **(B)** Under the same stimuli conditions, MPDs in 100 slightly different *Sik1* knockdown cells show a marked domain where singularity behavior may happen. Color bar indicates the MPD from 0 to 11 h. Similarly, the variances of the circadian phases in 100 WT cells **(C)** and *Sik1* knockdown cells **(D)** are also exhibited. Color bar indicates the variance from 0 to 18.

We have demonstrated that SIK1-related light adaptation can effectively reduce the phase-shifting effect of the circadian clock under a short-term stimulus and jet lag protocol. Now we want to figure out whether light adaptation can reduce or avoid singularity behavior. We recorded the behaviors of 100 cells’ response to a stimulus. The initial conditions for these 100 cells are slightly different. We first obtained the variable values of the circadian clock at CT0 in constant darkness. After that, for each variable, the initial condition is changed to the value at CT0 plus a random number between −5% and 5% of its amplitude. Thus, the 100 cells are randomly distributed in a small neighborhood of the point at CT0 on the stable periodic orbit. Since the desynchronization of the individual cellular clock is a key feature of singularity behavior, we measured the maximal phase difference (MPD) between cells and the variance of the phases 24 h after the stimulus, to indicate the level of desynchronization. Singularity behaviors are more likely to happen when the MPD and the variance of the phases of 100 cells are large. Singularity behavior only occurs within a small window of the stimulus time and stimulus strength (Huang et al., 2006). Thus, we first roughly scanned the stimulus time point from CT0h to CT24 h (interval of 0.5 h) and stimulus durations from 0 h to 7 h (interval of 0.5 h) to locate the area of singularity behavior (data not shown). Then, we refined the scan in the area of light pulse durations from 3h to 7 h (interval of 0.2 h) and light pulse beginning from CT14 h to CT19 h (interval of 0.2 h). The heatmap shown in Figure 7 describes the MPDs and variances of the phases in this area. The color bar on the right side of Figures 7A,B indicates the MPD from 0 h (blue) to 11 h (yellow). Compared with WT mice (Figure 7A), the phase variations in *Sik1* knockdown mice are much larger in a global picture (Figure 7B). The maximal MPD in *Sik1* knockdown mice is 10.61 h when the light pulse is applied at CT14.6 h for 7 h, while WT mice only show 3.5 h of the maximal phase shift. Similarly, the variances of the phases exhibit the same region where singularity is more likely to happen (Figures 7C,D).

Furthermore, we also performed the analysis of parameter disturbance when the light stimulus is applied at CT14.6 h for 7 h. The simulation results showed that 41.8% (957 out of 2,287) parameter sets can yield MPDs larger than 5 h for 100 *Sik1* knockdown cells. By contrast, only 23.2% (531 out of 2,287) parameter sets generate MPDs larger than 5 h for 100 WT cells. The situation is similar for the variances of the phases (Supplementary Figure S4). For 18.45% (422 out of 2,287) parameter sets, the differences of the variances are smaller than −5 (the variance in WT minus that in *Sik1* knockdown model). However, for only 4.63% (106 out of 2,287) parameter sets, the differences of the variances are larger than 5 (Supplementary Figure S4).These results indicate that the risk of singularity behavior is significantly increased in *Sik1* knockdown mice.

**FIGURE 8:**
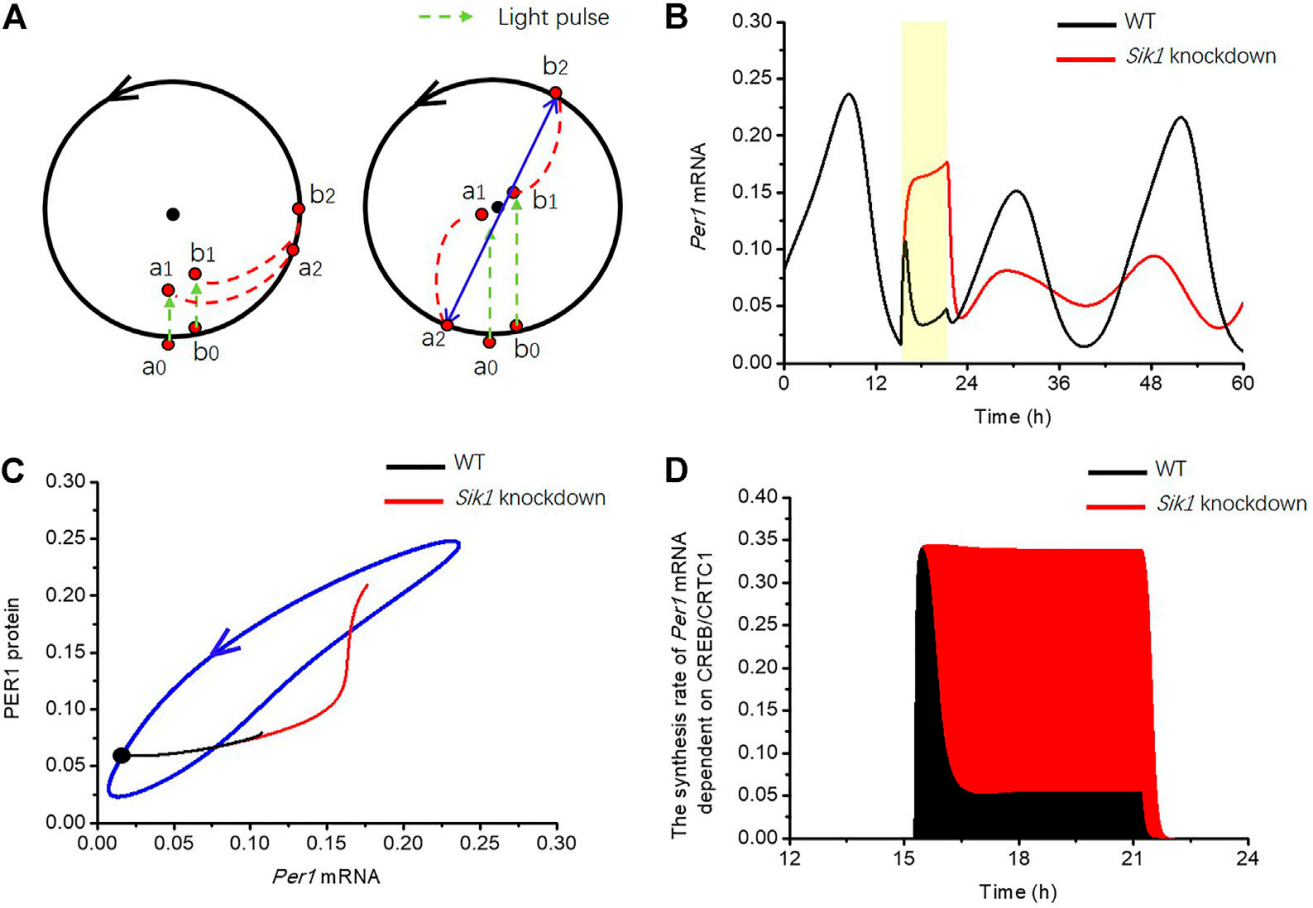
Mechanism by which light adaptation reduces the risk of singularity behavior in the circadian clock **(A)** Limit cycle model is used for the explanation of phase desynchronization. Black dot in the middle of the limit cycle is the unstable fixed point. (left panel) If two orbits starting from a_0_, b_0_, were pushed to a_1_, b_1_ by stimulus (green dashed arrow), the phase difference of these two trajectories is only slightly changed when the orbits returned to the limit cycle a_2_, b_2_. (right panel) However, if the stimulus is applied with appropriate strength at appropriate time, the two orbits are pushed to a small neighborhood of the unstable fixed point a_1_, b_1_. The trajectories may return to the limit cycle and reach markedly different phases. **(B)** Time series of *Per1* mRNA in WT (black curve) and *Sik1* knockdown mice (red curve) are exhibited when a 6-h light pulse (the yellow region) is given at CT15.2 h. **(C)** Related *Per1* mRNA—PER1 protein phase plane shows that this 6-h light pulse (from the black dot) pushes the orbit much further in *Sik1* knockdown mice (red) than that in WT (black), leading to the larger chance of crossing the singularity point. The blue closed curve corresponds to the oscillation of circadian clock in constant darkness. **(D)** Light adaptation significantly suppresses the synthesis rate of *Per1* mRNA in WT mice (black) upon light stimulation, so that the accumulation of *Per1* mRNA dependent on light (filled area) is much smaller than that in *Sik1* knockdown mice (red). Thus, the movement of *Per1* mRNA in the phase plane is restricted.

Why *Sik1* knockdown mice are more prone to singularity behavior? Again, let us explain the mechanism of singularity behavior by employing the simple limit-cycle model. As mentioned above, the limit cycle model has an attractive circular orbit and an unstable fixed point, which is called the “singularity point”. To illustrate the emergence of singularity behavior, the dynamics of the two points with slightly different phases, which are close to the limit cycle, are shown in Figure 8A (point a_0_, b_0_). If a light pulse was given at the appropriate time and with suitable strength, these two points could have been brought into a small neighborhood of the unstable fixed point but separated by it (right panel, point a_1_, b_1_). After the stimulus ends, the system returns to the limit cycle from these two initial conditions with totally different phases (right panel, point a_2_, b_2_). Therefore, the clocks in the two cells can be fully desynchronized (singularity behavior happens) when light stimulus is given at the appropriate time and with the appropriate magnitude (Figure 8A, right panel). If the stimulus time or strength is not appropriate, these two points cannot be brought to the neighborhood of the unstable fixed point, and the trajectories will return to the limit cycle with nearly the same phase, so that the cells will remain synchronized (Figure 8A, left panel).

Then, we returned to the coupled model to reveal the reason of light adaptation in reducing the risk of singularity behavior. We used the situation of 6-h light pulse at CT15.2 h as an example to analyze the singularity behavior. The time courses of *Per1* mRNA in WT and *Sik1* knockdown mice are shown in Figure 8B. By comparison with WT model, the concentration of *Per1* mRNA quickly increases and remains high during the light pulse in the situation of the *Sik1* knockdown model. In the phase plane of *Per1* mRNA-PER1 protein, the movement of *Per1* mRNA in the knockdown model of *Sik1* is much further than that in WT (Figure 8C). In the mammalian circadian system, it is usually hard to push a point from the stable cycle near or even cross the fixed point (Ukai et al., 2007). Hence, further movement of the point means a larger chance of crossing the singularity point. Therefore, the possibility of singularity occurrence in *Sik1* knockdown mice is larger than that in WT mice.

Furthermore, as we have mentioned above, the pushed distance of mRNA is determined by *Per1* mRNA accumulation upon the stimulus, which depends on the quantity of the nuclear CREB/CRTC1 complex. Because SIK1 inhibits the CREB/CRTC1 complex, the synthesis rate of light-induced *Per1* mRNA is reduced in the WT by the negative feedback loop early before the end of light pulse (Figure 8D). Thus, the accumulation of *Per1* mRNA due to light is much less than that in *Sik1* knockdown mice. To summarize, due to light adaption from the CRTC1-SIK1 pathway, the movement of *Per1* mRNA in the phase plane is reduced and it is hard to push the circadian orbit to the small neighborhood of the fixed point. As a consequence, singularity is less likely to occur in the WT condition.

### Light Adaptation Generates Refractoriness

Refractory behavior is a property common to many excitable and oscillatory systems, including the mammalian circadian clock which shows unresponsiveness to a second light stimulus. Here, in order to clarify the response of the mammalian circadian clock to frequent stimuli and the role of light adaptation, we considered the situation where the circadian clock is subjected to two successive light stimuli with short gap and simulated the CRTCI-SIK1 pathway in the WT and *Sik1* knockdown conditions. We assumed the formation of the CREB/CRTC1 complex (CC) as an indicator of light-induced response.

**FIGURE 9:**
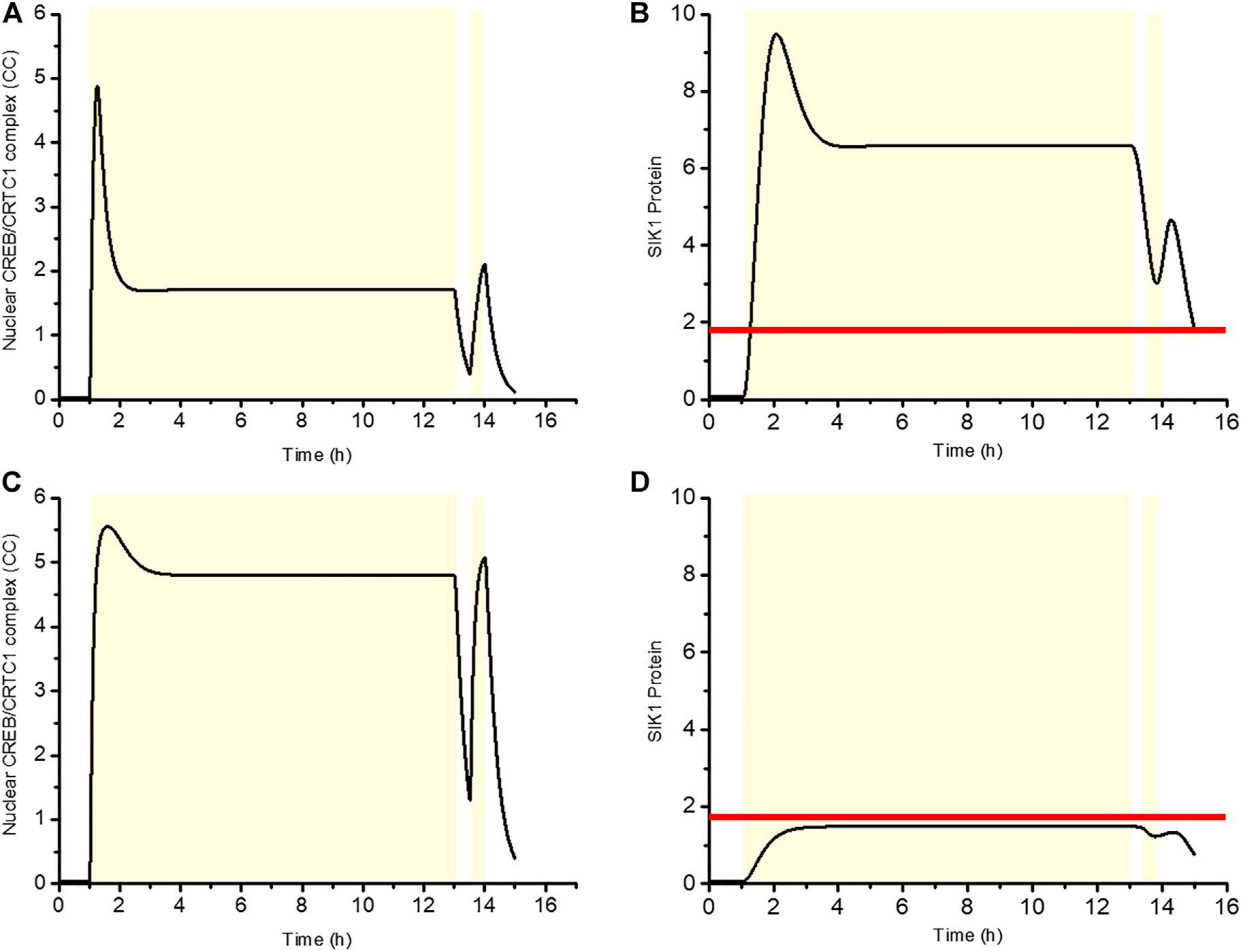
Negative regulation of SIK1 gives rise to refractoriness. The first light pulse is applied at 1 h for 12 h, and 30 min later, a second light stimulation is given for 30 min. The dynamics of the CREB/CRTC1 complex (CC) and SIK1 are shown in WT **(A, B)** and in *Sik1* knockdown conditions **(C, D)**. Yellow zone indicates the light stimulus. Horizontal line in **(B)** and **(D)** denotes the threshold of SIK1 that triggers the negative regulation. The horizontal line is determined by the function $\frac{SIK1^{m1\_SIK1}}{K1_{SIK1}^{m1\_SIK1}+SIK1^{m1\_SIK1}}$ , which represents the inhibition effect of SIK1 to nuclear CRTC1 (CRTC1_N_). When the inhibition effect equals 0.1, it corresponds to the threshold in **(B)** and **(D)**.

As is shown in Figure 2A, the phenomenon of light adaptation occurs when a constant light stimulation is given at 1 h. Then, after removing this light stimulation at 13 h and imposing 30 min of darkness, a new stimulus is applied for 30 min. Figures 9A,B exhibited the time series for the WT situation, where CC first increases rapidly and then reaches a plateau, while SIK1 follows a similar time course and stabilizes at a high plateau level. During the second stimulus, CC increases gradually, but the rise in CC remains reduced, because SIK1 is still at a high level. Therefore SIK1 suppresses the CC response to the second stimulus. By contrast, in the knockdown condition, CC increases rapidly after the first stimulus and also following the second stimulus, because the level of SIK1 remains low (Figures 9C,D). These results indicate that the CC response in the WT situation displays refractoriness, in contrast to the behavior in knockdown condition. In both Figures 9B,D the horizontal line represents the threshold of SIK1 that triggers the negative feedback responsible for adpatation and refractoriness. As long as the system remains above this threshold, light stimuli are not able to elicit a response, which reflects the existence of a refractory period.

In conclusion, the CRTC1-SIK1 pathway underlies light adaptation of the circadian clock, and, at the same time, allows refractory behavior by which the system is able to resist the frequent external changes that likely characterize light stimuli in the natural environment.

## Discussion

Generally, adaptation is a phenomenon characterized by the ability of circuits to produce a response to some input change, which decreases even when the input change persists. This mechanism is crucial since physiological systems usually need to buffer the large fluctuations from the external environment. All possible three-node network topologies which could perform adaptation have been investigated by Chao Tang et al (Ma et al., 2009). This research found two major core topologies emerging as a robust solution: a negative feedback loop with a buffering node and an incoherent feedforward loop with a proportioner node (Figure 3 in the reference (Ma et al., 2009)). In fact, an excellent biochemical system not only has adaptation but also has sensitivity, which is defined as the height of the output response relative to the initial steady-state value (Koshland et al., 1982). The sensitivity of the biochemical system helps the system to quickly sense and respond to changing environmental conditions.

Experimental results have shown that the circadian genes can respond to the light stimulus rapidly. And this effect is attenuated to avoid excessive changes of the circadian clock, in order to adapt to the varied environment smoothly. For instance, circadian clocks can be entrained to the natural LD cycle. Nevertheless, the adjustment of the circadian clock to the external environment is slow. Take the experience of the jet-lag protocol, for example, it usually takes roughly *n* days to adapt to a phase shift of *n* hours after a long-distance flight. Recent experimental advances have shed light on the molecular mechanism that can limit the effects of light stimuli in circadian clocks. The study of Jagganath et al. thus showed that a key role in light adaptation is played by the CRTC1-SIK1 pathway (Jagannath et al., 2013), which introduces a negative feedback loop that suppresses the effect of light on the circadian clock.

In this study, based on the experimental data (Jagannath et al., 2013), we built and simulated a mathematical model for light adaptation of the circadian system via the CRTC1-SIK1 pathway. We first studied the response of the isolated CRTC1-SIK1 module to light stimulus, and found that the circadian clock showed the characteristics of light adaptation in constant light. The light adaptation allows the circadian clock to respond to light changes rapidly and to diminish this response under constant stimulation. The model provides a detailed quantitative explanation for the occurrence of light adaptation and shows how the negative feedback loop provided by SIK1 generates light adaptation for the circadian clock. We then coupled the CRTC1-SIK1 model to an existing model of the cell-autonomous mammalian circadian clock (Mirsky et al., 2009). This coupled model is employed to explore the roles of light adaptation in the mammalian circadian clock. We revealed that light adaptation favors the phase robustness of the circadian clock. The simulation results showed that the effect of the phase shift is limited upon a short-term light pulse and a jet-lag experiment, which matches the experimental observation. The underlying reason for the phase robustness is explained by the phase plane of the *Per1* mRNA-PER1 protein. Furthermore, by extensive numerical simulations, we also predicted that light adaptation reduces the likelihood of singularity behavior. The mechanism underlying this reduction was analyzed with the help of an abstract limit-cycle model. The light adaptation provided by the CRTC1-SIK1 pathway suppresses the continuous response to the light pulse, and thereby reduces the stimulus-induced displacement of *Per* mRNAs away from the point on the limit cycle at which the system is perturbed. By contrast, in the absence of adaptation, pertubations given at the appropriate phase with the right magnitude are able to bring the points from the limit cycle close to the singularity point from where the trajectories return to the limit cycle and reach it at different phases; this results in the suppression of the average periodic behavior of the clocks. Therefore, light adaptation can significantly reduce the risk of singularity behavior. Finally, our simulations showed that after an extended exposure to light, the circadian clock displays refractoriness to an additional stimulus. After the first stimulus, the level of SIK1 rises, indeed, above a threshold value so that the response to a second stimulus is suppressed during a certain time window.

Some previous studies have proved that the coupling between cells is also a critical factor for the synchronization of circadian clocks, preventing the occurrence of singularity (Ukai et al., 2007; Abraham et al., 2010). The phase response curves showed that the average magnitude of cellular phase shifts in *in vitro* experiments is relatively larger than that of organismal phase shifts. It might be attributed to the coupling mechanism of multiple individual clock cells. This coupling mechanism provides a different way to avoid the desynchronization through cell–cell communication. The mathematical model used in the present study does not include intercellular interactions. We focused on the intrinsic regulatory mechanism of individual cells and proposed the hypothesis that the CRTC1-SIK1 module prevents singularity by avoiding excessive change of circadian genes. This mechanism works inside individual cells, which has not been studied experimentally. In the future research studies, it would be worth to explore if the mechanisms of dual protection from singularity behavior can further enhance the robustness of the circadian clock to the deleterious fluctuations of the external environment.

n conclusion, the model shows that light adaptation attenuates the induction of clock genes in response to light. By enhancing the phase robustness of the oscillations and reducing the risk of deleterious singularity behavior, the adaptation pathway allows the circadian system to be shielded from the disruptions due to fluctuations in the light input. Moreover, light adaptation controlled by SIK1 plays an important role in maintaining homeostasis of the circadian clock with regard to the fluctuations of the natural environment.

## Data Availability

The original contributions presented in the study are included in the article/Supplementary Material; further inquiries can be directed to the corresponding author.
