# Supplementary material for: A Mathematical Model to Characterize the Role of Light Adaptation in Mammalian Circadian Clock
Source: Front Mol Biosci. 2021 Dec 7;8:681696. doi: 10.3389/fmolb.2021.681696 (PMC8691188; doi:10.3389/fmolb.2021.681696)
Supplement: Supplementary file 1 [file DataSheet1.pdf]

Supplementary Material

1 Kinetic equations for the CRTC1-SIK1 pathway and for its coupling to the circadian clock

The molecular processes can be described as the following ordinary differential equations. Here the symbols indicate the concentrations of the elements in the model. The ordinary differential equations for the CRTC1-SIK1 pathway are listed as follows:

$$\frac{dCRTC1_{PC}}{dt} = K_{pc\_x}CRTC1_C + \frac{K_{pex\_x}CRTC1_{PN}}{a} - \left(V_{0\_x} + L\right)CRTC1_{PC} - K_{pim\_x}CRTC1_{PC} \quad (1)$$

$$\frac{dCRTC1_C}{dt} = \left(V_{0\_x} + L\right)CRTC1_{PC} + \frac{K_{ex\_x}CRTC1_N}{a} - K_{pc\_x}CRTC1_C - K_{im\_x}CRTC1_C \quad (2)$$

$$\frac{dCRTC1_N}{dt} = aK_{im\_x}CRTC1_C + K_{dp\_x}CRTC1_{PN} - K_{ex\_x}CRTC1_N - \left(a1 + \frac{b1 \cdot SIK1^{m1\_SIK1}}{K1_{SIK1}^{m1\_SIK1} + SIK1^{m1\_SIK1}}\right)CRTC1_N + km_{CC} \cdot CC - V_{0\_CC}CRTC1_N CREB_{PN} \quad (3)$$

$$CRTC1_{PN} = tot - CRTC1_N - a(CRTC1_{PC} + CRTC1_C) - CC \quad (4)$$

$$\frac{dCREB_{PN}}{dt} = K_{pn\_y}CREB_N + a \cdot K_{pim\_y}CREB_{PC} - K_{pex\_y}CREB_{PN} - K_{dpn\_y}CREB_{PN} + (km_{CC} \cdot CC - V_{0\_CC}CREB_{PN}CRTC1_N) \quad (5)$$

$$\frac{dCREB_{PC}}{dt} = \left(V_{0\_y} + 0.8L\right)CREB_C + \frac{K_{pex\_y} \cdot CREB_{PN}}{a} - K_{pim\_y}CREB_{PC} - K_{dp\_y}CREB_{PC} \quad (6)$$

$$\frac{dCREB_C}{dt} = \frac{K_{ex\_y}CREB_N}{a} + K_{dp\_y}CREB_{PC} - \left(V_{0\_y} + 0.8L\right)CREB_C - K_{im\_y}CREB_C \quad (7)$$

$$CREB_{PN} = tot - CREB_N - a(CREB_{PC} + CREB_C) - CC \quad (8)$$

$$\frac{dCC}{dt} = V_{0\_CC}CRTC1_N CREB_{PN} - km_{CC}CC \quad (9)$$

$$\frac{dmSik}{dt} = \left(V0_{sik1} + V1_{sik1}\frac{CC^{m1\_CC}}{K1_{CC}^{m1\_CC} + CC^{m1\_CC}}\right) - km_{sik1}mSik1 \quad (10)$$

$$\frac{dSIK1}{dt} = V0_{SIK1}mSik1 - km_{SIK1}SIK1 \quad (11)$$

For the mammalian circadian clock, we listed all the 21 differential equations, eq(12)- eq(32), based on the model which is developed by Henry P. Mirsky et al (Mirsky et al., 2009, supporting information). Experimental results have shown that the nuclear CREB/CRTC1 complex can up-regulate the expressions of *Per1* and *Per2* mRNA by

binding to the CRE elements in their promoters (Jagannath et al., 2013). Therefore, to couple the CRTC1/SIK1 pathway to the mammalian circadian clock, we added a term which is dependent on the concentration of nuclear CREB/CRTC1 complex (CC) in the equations for the time evolutions of *Per1* mRNA and *Per2* mRNA .

$$\frac{dmPer1}{dt} = \left(V0_{P1} + V1_{P1}\frac{CLK\_BMAL1^{na1\_P1}}{KA1_{P1}{}^{na1\_P1}+CLK\_BMAL1^{na1\_P1}}\right)\cdot\frac{KI1_{P1}1^{ni1\_P1}}{KI1_{P1}{}^{ni1\_P1}+PER1\_CRY1^{ni1\_P1}}\cdot\frac{KI2_{P1}{}^{ni2\_P1}}{KI2_{P1}{}^{ni2\_P1}+PER1\_CRY2^{ni2\_P1}}\cdot\frac{KI3_{P1}{}^{ni3\_P1}}{KI3_{P1}{}^{ni3\_P1}+PER2\_CRY1^{ni3\_P1}}\cdot\frac{KI4_{P1}{}^{ni4\_P1}}{KI4_{P1}{}^{ni4\_P1}+PER2\_CRY2^{ni4\_P1}} + V2_{P1}\cdot\frac{CC^{m2\_CC}}{K2_{CC}{}^{m2\_CC}+CC^{m2\_CC}} - km_{P1}\cdot mPer1 \quad (12)$$

$$\frac{dmPer2}{dt} = (\,V0_{P2} + V1_{P2}\frac{CLK\_BMAL1^{na1\_P2}}{KA1_{P2}{}^{na1\_P2}+CLK\_BMAL1^{na1\_P2}})\cdot\frac{KI1_{P2}{}^{ni1\_P2}}{KI1_{P2}{}^{ni1\_P2}+PER1\_CRY1^{ni1\_P2}}\cdot\frac{KI2_{P2}{}^{ni2\_P2}}{KI2_{P2}{}^{ni2\_P2}+PER1\_CRY2^{ni2\_P2}}\cdot\frac{KI3_{P2}{}^{ni3\_P2}}{KI3_{P2}{}^{ni3\_P2}+PER2\_CRY1^{ni3\_P2}}\cdot\frac{KI4_{P2}{}^{ni4\_P2}}{KI4_{P2}{}^{ni4\_P2}+PER2\_CRY2^{ni4\_P2}} + V2_{P2}\frac{CC^{m3\_CC}}{K3_{CC}{}^{m3\_CC}+CC^{m3\_CC}} - km_{P2}\cdot mPer2 \quad (13)$$

$$\frac{dmCry1}{dt} = \left(V0_{C1} + V1_{C1}\frac{CLK\_BMAL1^{na1\_C1}}{KA1_{C1}{}^{na1\_C1}+CLK\_BMAL1^{na1\_C1}} + V2_{C1}\frac{RORc^{na2\_C1}}{KA2_{C1}{}^{na2\_C1}+RORc^{na2\_C1}}\right)\cdot\frac{KI1_{C1}{}^{ni1\_C1}}{KI1_{C1}{}^{ni1\_C1}+PER1\_CRY1^{ni1\_C1}}\cdot\frac{KI2_{C1}{}^{ni2\_C1}}{KI2_{C1}{}^{ni2\_C1}+PER1\_CRY2^{ni2\_C1}}\cdot\frac{KI3_{C1}{}^{ni3\_C1}}{KI3_{C1}{}^{ni3\_C1}+PER2\_CRY1^{ni3\_C1}}\cdot\frac{KI4_{C1}{}^{ni4\_C1}}{KI4_{C1}{}^{ni4\_C1}+PER2\_CRY2^{ni4\_C1}}\cdot\frac{KI5_{C1}{}^{ni5\_C1}}{KI5_{C1}{}^{ni5\_C1}+REV\_ERB^{ni5\_C1}} - km_{C1}\cdot mCry1 \quad (14)$$

$$\frac{dmCry2}{dt} = (V0_{C2} + V1_{C2}\frac{CLK\_BMAL1^{na1\_C2}}{KA1_{C2}{}^{na1\_C2}+CLK\_BMAL1^{na1\_C2}} + V2_{C2}\frac{RORc^{na2\_C2}}{KA2_{C2}{}^{na2\_C2}+RORc^{na2\_C2}})\cdot\frac{KI1_{C2}{}^{ni1\_C2}}{KI1_{C2}{}^{ni1\_C2}+PER1\_CRY1^{ni1\_C2}}\cdot\frac{KI2_{C2}{}^{ni2\_C2}}{KI2_{C2}{}^{ni2\_C2}+PER1\_CRY2^{ni2\_C2}}\cdot\frac{KI3_{C2}{}^{ni3\_C2}}{KI3_{C2}{}^{ni3\_C2}+PER2\_CRY1^{ni3\_C2}}\cdot\frac{KI4_{C2}{}^{ni4\_C2}}{KI4_{C2}{}^{ni4\_C2}+PER2\_CRY2^{ni4\_C2}}\cdot\frac{KI5_{C2}{}^{ni5\_C2}}{KI5_{C2}{}^{ni5\_C2}+REV\_ERB^{ni5\_C2}} - km_{C2}\cdot mCry2 \quad (15)$$

$$\frac{dmRev\_erb\alpha}{dt} = V1_{Re}\frac{CLK\_BMAL1^{na1\_Re}}{KA1_{Re}{}^{na1\_Re}+CLK\_BMAL1^{na1\_Re}}\cdot\frac{KI1_{Re}{}^{ni1_{Re}}}{KI1_{Re}{}^{ni1_{Re}}+PER1_{CRY_1}{}^{ni1_{Re}}}\cdot\frac{KI2_{Re}{}^{ni2_{Re}}}{KI2_{Re}{}^{ni2_{Re}}+PER1_{CRY_2}{}^{ni2_{Re}}}\cdot\frac{KI3_{Re}{}^{ni3_{Re}}}{KI3_{Re}{}^{ni3_{Re}}+PER2_{CRY_1}{}^{ni3_{Re}}}\cdot\frac{KI4_{Re}{}^{ni4_{Re}}}{KI4_{Re}{}^{ni4_{Re}}+PER2_{CRY_2}{}^{ni4_{Re}}} - km_{Re}\cdot mRev\_erb\alpha \quad (16)$$

$$\frac{dmClk}{dt} = (V0_{Clk} + V1_{Clk}\frac{RORc^{na1\_Clk}}{RORc^{na1\_Clk}+KA1_{Clk}{}^{na1\_Clk}})\cdot\frac{KI1_{Clk}{}^{ni1\_Clk}}{REV\_ERB^{ni1\_Clk}+KI1_{Clk}{}^{ni1\_Clk}} - km_{Clk}\cdot mClk \quad (17)$$

$$\frac{dmBmal1}{dt} = (V0_{Bmal1} + V1_{Bmal1}\frac{RORc^{na1\_Bmal1}}{KA1_{Bmal1}{}^{na1\_Bmal1}+RORc^{na1\_Bmal1}})\cdot\frac{KI1_{Bmal1}{}^{ni1\_Bmal1}}{REV\_ERB^{ni1\_Bmal1}+KI1_{Bmal1}{}^{ni1\_Bmal1}} - km_{Bmal1}\cdot mBmal1 \quad (18)$$

$$\frac{dmRorc}{dt} = (V0_{Rorc} + V1_{Rorc} \frac{CLK\_BMAL1^{na1\_Rorc}}{CLK\_BMAL1^{na1\_Rorc} + KA1_{Rorc}{}^{na1\_Rorc}} + V2_{Rorc} \frac{RORc^{na2\_Rorc}}{KA2_{Rorc}{}^{na2\_Rorc} + RORc^{na2\_Rorc}}) \cdot \frac{KI1_{Rorc}{}^{ni1\_Rorc}}{KI1_{Rorc}{}^{ni1\_Rorc} + PER1\_CRY1^{ni1\_Rorc}} \cdot \frac{KI2_{Rorc}{}^{ni2\_Rorc}}{KI2_{Rorc}{}^{ni2\_Rorc} + PER1\_CRY2^{ni2\_Rorc}} \cdot \frac{KI3_{Rorc}{}^{ni3\_Rorc}}{KI3_{Rorc}{}^{ni3\_Rorc} + PER2\_CRY1^{ni3\_Rorc}} \cdot \frac{KI4_{Rorc}{}^{ni4\_Rorc}}{KI4_{Rorc}{}^{ni4\_Rorc} + PER2\_CRY2^{ni4\_Rorc}} \cdot \frac{KI5_{Rorc}{}^{ni5\_Rorc}}{KI5^{ni5\_Rorc} + REV\_ERB^{ni5\_Rorc}} - km_{Rorc} \cdot mRorc \quad (19)$$

$$\frac{dPER1}{dt} = t\_P1 \cdot mPer1 - a\_P1\_C1 \cdot PER1 \cdot CRY1 - a\_P1\_C2 \cdot PER1 \cdot CRY2 + d\_P1\_C1 \cdot PER1\_CRY1 + d\_P1\_C2 \cdot PER1\_CRY2 - kp\_P1 \cdot PER1 \quad (20)$$

$$\frac{dPER2}{dt} = t\_P2 \cdot mPer2 - a\_P2\_C1 \cdot PER2 \cdot CRY1 - a\_P2\_C2 \cdot PER2 \cdot CRY2 + d\_P2\_C1 \cdot PER2\_CRY1 + d\_P2\_C2 \cdot PER2\_CRY2 - kp\_P2 \cdot PER2 \quad (21)$$

$$\frac{dCRY1}{dt} = t\_C1 \cdot mCry1 - a\_P1\_C1 \cdot PER1 \cdot CRY1 - a\_P2\_C1 \cdot PER2 \cdot CRY1 + d\_P1\_C1 \cdot PER1\_CRY1 + d\_P2\_C1 \cdot PER2\_CRY1 - kp\_C1 \cdot CRY1 \quad (22)$$

$$\frac{dCRY2}{dt} = t\_C2 \cdot mCry2 - a\_P1\_C2 \cdot PER1 \cdot CRY2 - a\_P2\_C2 \cdot PER2 \cdot CRY2 + d\_P1\_C2 \cdot PER1\_CRY2 + d\_P2\_C2 \cdot PER2\_CRY2 - kp\_C2 \cdot CRY2 \quad (23)$$

$$\frac{dREV\_ERB\alpha}{dt} = t_{REV_{ERB}} \cdot mRev\_erb\alpha - kp\_REV\_ERB \cdot REV\_ERB \quad (24)$$

$$\frac{dCLK}{dt} = t\_CLK \cdot mClk - a\_CLK\_BMAL1 \cdot CLK \cdot BMAL1 + d\_CLK\_BMAL1 \cdot CLK\_BMAL1 - kp\_CLK \cdot CLK \quad (25)$$

$$\frac{dBMAL1}{dt} = t\_Bmal1 \cdot mBmal1 - a\_CLK\_BMAL1 \cdot CLK \cdot BMAL1 + d\_CLK\_BMAL1 \cdot CLK\_BMAL1 - kp\_BMAL1 \cdot BMAL1 \quad (26)$$

$$\frac{dRORc}{dt} = t\_Rorc \cdot mRorc - kp\_RORc \cdot RORc \quad (27)$$

$$\frac{dPER1\_CRY1}{dt} = a\_P1\_C1 \cdot PER1 \cdot CRY1 - d\_P1\_C1 \cdot PER1\_CRY1 \quad (28)$$

$$\frac{dPER2\_CRY1}{dt} = a\_P2\_C1 \cdot PER2 \cdot CRY1 - d\_P2\_C1 \cdot PER2\_CRY1 \quad (29)$$

$$\frac{dPER1\_CRY2}{dt} = a\_P1\_C2 \cdot PER1 \cdot CRY2 - d\_P1\_C2 \cdot PER1\_CRY2 \quad (30)$$

$$\frac{dPER2\_CRY2}{dt} = a\_P2\_C2 \cdot PER2 \cdot CRY2 - d\_P2\_C2 \cdot PER2\_CRY2 \quad (31)$$

$$\frac{dCLK\_BMAL1}{dt} = a\_CLK\_BMAL1 \cdot CLK \cdot BMAL1 - d\_CLK\_BMAL1 \cdot CLK\_BMAL1 \quad (32)$$

The symbols of variables in the model are listed in Table S1. Table S2 and Table S3 exhibit the information of parameters (definitions, parameter symbols and parameter values) for the CRTC1-SIK1 model and mammalian circadian clock, respectively.

Table S1: Symbols of the variables for the CRTC1-SIK1 model and circadian clock model

Symbols	Variables
$CRTC1_C$	CRTC1 in the cytoplasm
$CRTC1_{PC}$	Phosphorylated CRTC1 in the cytoplasm
$CRTC1_N$	CRTC1 in the nucleus
$CRTC1_{PN}$	Phosphorylated CRTC1 in the nucleus
$CREB_C$	CREB in the cytoplasm
$CREB_{PC}$	Phosphorylated CREB in the cytoplasm
$CREB_N$	CREB in the nucleus
$CREB_{PN}$	Phosphorylated CREB in the nucleus
CC	Nuclear CRTC1 and CREB complex
$mPer1$	*Per1* mRNA
$mPer2$	*Per2* mRNA
$mCry1$	*Cry1* mRNA
$mCry2$	*Cry2* mRNA
$mRev\_erb\alpha$	*Rev-erb* α mRNA
$mClk$	*Clock* mRNA
$mBmal1$	*Bmal1* mRNA
$mRorc$	*Rorc* mRNA
$PER1$	PER1 protein
$PER2$	PER2 protein
$CRY1$	CRY1 Protein
$CRY2$	CRY2 Protein
$REV\_ERB\alpha$	REV-ERBα Protein
CLK	CLOCK Protein
$BMAL1$	BMAL1 Protein
$RORc$	RORc Protein
$PER1\_CRY1$	PER1/CRY1 complex
$PER1\_CRY2$	PER1/CRY2 complex
$PER2\_CRY1$	PER2/CRY1 complex
$PER2\_CRY2$	PER2/CRY2 complex
CLK_BMAL1	CLOCK/BMAL1 complex

The main purpose of the CRTC1-SIK1 model is to qualitatively analyze the dynamical mechanism of light adaptation. Therefore, the parameter values for the CRTC1-SIK1 model are chosen based on the qualitative change of experimental observations: (1) The self-inhibition of SIK1 should take effect. (2) The output of the CRTC1-SIK1 module doesn't not disrupt the oscillation of circadian clock.

Table S2: The definitions and values of the model for the CRTC1-SIK1 pathway

**Parameter definitions**	**Parameter symbol**	**Para meter value**
The basic dephosphorylation rate of cytoplasmic CRTC1	V_{0_x}	0.1
The phosphorylation rate of cytoplasmic CRTC1	K_{pc_x}	4
The transfer rate of dephosphorylated cytoplasmic CRTC1 into the nucleus	K_{pim_x}	0.5
The transfer rate of dephosphorylated nuclear CRTC1 into the cytoplasm	K_{pex_x}	100
The transfer rate of cytoplasmic CRTC1 into the nucleus	K_{im_x}	50
The transfer rate of nuclear CRTC1 into the cytoplasm	K_{ex_x}	1
The dephosphorylation rate of nuclear CRTC1	K_{dp_x}	1
The basic phosphorylation rate of nuclear CRTC1	$a1$	4.5
The basic phosphorylation rate of cytoplasmic CREB	V_{0_y}	0.5
The dephosphorylation rate of cytoplasmic CREB	K_{dp_y}	3
The transfer rate of phosphorylated CREB into the nucleus	K_{pim_y}	51.5
The transfer rate of phosphorylated CREB into the cytoplasm	K_{pex_y}	2
The transfer rate of CREB into the nucleus	$\mathrm{K_{im\_y}}$	0.1
The transfer rate of CREB transfer into the cytoplasm	K_{ex_y}	100
The phosphorylation rate of nuclear CREB	K_{pn_y}	1
The dephosphorylation rate of nuclear CREB	K_{dpn_y}	30
The association rate for CREB/CRTC1 complex	V_{0_CC}	3.2
The disassociation rate of CREB/CRTC1 complex	km_{CC}	3
The basic transcription rate of *Sik1*	$V0_{sik}$	0.1
The translation rate for SIK1 protein	$V0_{SIK}$	6.5
The degradation rate of *Sik1* mRNA	km_{sik1}	3
The degradation rate of SIK1 protein	km_{SIK1}	3
The maximal phosphorylation rate of nuclear CRTC1 dependent on SIK1	$b1$	250
The maximal transcriptional rate of Sik1 dependent on CREB/CRTC1 complex	$V1_{sik}$	20
The maximal transcriptional rate of *Per1* dependent on CREB/CRTC1 complex	$V2_{P1}$	0.35
The maximal transcriptional rate of *Per2* dependent on CREB/CRTC1 complex	$V2_{P2}$	0.04
Michaelis constant for nuclear CRTC1 phosphorylation due to SIK1 promotion	$K1_{SIK1}$	3
Michaelis constant for *Sik1* transcription due to CREB/CRTC1 complex	$K1_{CC}$	1.8
Michaelis constant for *Per1* transcription due to CREB/CRTC1 complex	$K2_{CC}$	2.4

Michaelis constant for *Per2* transcription due to CREB/CRTC1 complex	$K3_{CC}$	1.8
Hill coefficient for nuclear CRTC1 phosphorylation due to SIK1 promotion	$m1\_SIK1$	4
Hill coefficient for *Sik1* transcription due to CREB/CRTC1 complex	$m1\_CC$	4
Hill coefficient for *Per1* transcription due to CREB/CRTC1 complex	$m2\_CC$	5
Hill coefficient for *Per2* transcription due to CREB/CRTC1 complex	$m3\_CC$	4
Total amounts of CRTC1 and CREB	tot	10
The light effect	L	40
The volume ratio between the cytoplasm and nucleus	a	10

Table S3: The definitions and values for the model of the mammalian circadian clock

**Parameter name**	**Parameter symbol**	**Parameter value**
Basal rate of *mPer1* transcription	$V0_{P1}$	0.17nM/h
Maximum rate of *mPer1* transcription from *CLK_BMAL1* activation	$V1_{P1}$	3.84nM/h
Basal rate of *mPer2* transcription	$V0_{P2}$	0.09nM/h
Maximum rate of *mPer2* transcription from *CLK_BMAL1* activation	$V1_{P2}$	3.29nM/h
Basal rate of *mCry1* transcription	$V0_{C1}$	0.26nM/h
Maximum rate of *mCry1* transcription from CLK_BMAL1 activation	$V1_{C1}$	2.44nM/h
Maximum rate of *mCry*1 transcription from *RORc* activation	$V2_{C1}$	2.89nM/h
Basal rate of *mCry2* transcription	$V0_{C2}$	1.29nM/h
Maximum rate of *mCry2* transcription from *CLK_BMAL1* activation	$V1_{C2}$	2.72nM/h
Maximum rate of *mCry2* transcription from *RORc* activation	$V2_{C2}$	4.28nM/h
Maximum rate of *REV_ERBα* transcription from *CLK_BMAL1* activation	$V1_{Re}$	1.03nM/h
Basal rate of *mClk* transcription	$V0_{Clk}$	3.98nM/h
Maximum rate of *mClk* transcription from RORc activation	$V1_{Clk}$	3.36nM/h
Basal rate of *mBmal1* transcription	$V0_{Bmal1}$	1.98nM/h
Maximum rate of *mBmal1* transcription from RORc activation	$V1_{Bmal1}$	4.12nM/h
Basal rate of *mRorc* transcription	$V0_{Rorc}$	0.69nM/h

Maximum rate of *mRorc* transcription from *CLK_BMAL1* activation	$V1_{Rorc}$	3.55nM/h
Maximum rate of *mRorc* transcription from *RORc* activation	$V2_{Rorc}$	0.46nM/h
Hill coeffificient for *mPer1* transcription due to *CLK_BMAL1* activation	*na1_P1*	3.28
Hill coeffificient for *mPer1* transcription due to *PER1_CRY1* inhibition	*ni1_P1*	3.93
Hill coeffificient for *mPer1* transcription due to *PER1_CRY2* inhibition	*ni2_P1*	2.61
Hill coeffificient for *mPer1* transcription due to *PER2_CRY1* inhibition	*ni3_P1*	1.66
Hill coeffificient for *mPer1* transcription due to *PER2_CRY2* inhibition	*ni4_P1*	4.85
Hill coeffificient for *mPer2* transcription due to *CLK_BMAL1* activation	*na1_P2*	4.50
Hill coeffificient for *mPer2* transcription due to *PER1_CRY1* inhibition	*ni1_P2*	4.45
Hill coeffificient for *mPer2* transcription due to *PER1_CRY2* inhibition	*ni2_P2*	3.70
Hill coeffificient for *mPer2* transcription due to *PER2_CRY1* inhibition	*ni3_P2*	4.35
Hill coeffificient for *mPer2* transcription due to *PER2_CRY2* inhibition	*ni4_P2*	3.67
Hill coeffificient for *mCry1* transcription due to *CLK_BMAL1* activation	*na1_C1*	4.91
Hill coeffificient for *mCry1* transcription due to *RORc* activation	*na2_C1*	3.01
Hill coeffificient for *mCry1* transcription due to *PER1_CRY1* inhibition	*ni1_C1*	4.65
Hill coeffificient for *mCry1* transcription due to *PER1_CRY2* inhibition	*ni2_C1*	3.66
Hill coeffificient for *mCry1* transcription due to *PER2_CRY1* inhibition	*ni3_C1*	2.59
Hill coeffificient for *mCry1* transcription due to *PER2_CRY2* inhibition	*ni4_C1*	0.29
Hill coeffificient for *mCry1* transcription due to *REV_ERB*α inhibition	*ni5_C1*	2.24
Hill coeffificient for *mCry2* transcription due to *CLK_BMAL1* activation	*na1_C2*	4.39
Hill coeffificient for *mCry2* transcription due to *RORc* activation	*na2_C2*	4.43
Hill coeffificient for *mCry2* transcription due to *PER1_CRY1* inhibition	*ni1_C2*	4.68

Hill coeffificient for *mCry2* transcription due to *PER1_CRY2* inhibition	*ni2_C2*	4.71
Hill coeffificient for *mCry2* transcription due to *PER2_CRY1* inhibition	*ni3_C2*	2.23
Hill coeffificient for *mCry2* transcription due to *PER2_CRY2* inhibition	*ni4_C2*	0.48
Hill coeffificient for *mCry2* transcription due to *REV_ERB*α inhibition	*ni5_C2*	1.75
Hill coeffificient for *mRev_erb*α transcription due to *CLK_BMAL1* activation	*na1_Re*	4.40
Hill coeffificient for *mRev_erb*α transcription due to *PER1_CRY1* inhibition	*ni1_Re*	0.30
Hill coeffificient for *mRev_erb*α transcription due to *PER1_CRY2* inhibition	*ni2_Re*	0.67
Hill coeffificient for *mRev_erb*α transcription due to *PER2_CRY1* inhibition	*ni3_Re*	1.93
Hill coeffificient for *mRev_erb*α transcription due to *PER2_CRY2* inhibition	*ni4_Re*	3.58
Hill coeffificient for *mClk* transcription due to *RORc* activation	*na1_Clk*	3.50
Hill coeffificient for *mClk* transcription due to *REV_ERB*α inhibition	*ni1_Clk*	1.96
Hill coeffificient for *mBmal1* transcription due to *RORc* activation	*na1_Bmal1*	4.13
Hill coeffificient for *mBmal1* transcription due to *REV_ERB*α inhibition	*ni1_Bmal1*	0.02
Hill coeffificient for *mRorc* transcription due to *CLK_BMAL1* activation	*na1_Rorc*	1.57
Hill coeffificient for *mRorc* transcription due to *RORc* activation	*na2_Rorc*	0.56
Hill coeffificient for *mRorc* transcription due to *PER1_CRY1* inhibition	*ni1_Rorc*	2.25
Hill coeffificient for *mRorc* transcription due to *PER1_CRY2* inhibition	*ni2_Rorc*	4.73
Hill coeffificient for *mRorc* transcription due to *PER2_CRY1* inhibition	*ni3_Rorc*	3.43
Hill coeffificient for *mRorc* transcription due to *PER2_CRY2* inhibition	*ni4_Rorc*	0.84
Hill coeffificient for *mRorc* transcription due to *REV_ERB*α inhibition	*ni5_Rorc*	4.33
Michaelis constant for *mPer1* transcription due to *CLK_BMAL1* activation	$KA1_{P1}$	1.98nM

Michaelis constant for *mPer1* transcription due to *PER1_CRY1* inhibition	$KI1_{P1}$	1.07nM
Michaelis constant for *mPer1* transcription due to *PER1_CRY2* inhibition	$KI2_{P1}$	3.96nM
Michaelis constant for *mPer1* transcription due to *PER2_CRY1* inhibition	$KI3_{P1}$	1.68nM
Michaelis constant for *mPer1* transcription due to *PER2_CRY2* inhibition	$KI4_{P1}$	3.11nM
Michaelis constant for *mPer2* transcription due to *CLK_BMAL1* activation	$KA1_{P2}$	1.90nM
Michaelis constant for *mPer2* transcription due to *PER1_CRY1* inhibition	$KI1_{P2}$	4.51nM
Michaelis constant for *mPer2* transcription due to *PER1_CRY2* inhibition	$KI2_{P2}$	2.98nM
Michaelis constant for *mPer2* transcription due to *PER2_CRY1* inhibition	$KI3_{P2}$	2.24nM
Michaelis constant for *mPer2* transcription due to *PER2_CRY2* inhibition	$KI4_{P2}$	3.31nM
Michaelis constant for *mCry1* transcription due to *CLK_BMAL1* activation	$KA1_{C1}$	1.46nM
Michaelis constant for *mCry1* transcription due to *RORc* activation	$KA2_{C1}$	3.76nM
Michaelis constant for *mCry1* transcription due to *PER1_CRY1* inhibition	$KI1_{C1}$	0.03nM
Michaelis constant for *mCry1* transcription due to *PER1_CRY2* inhibition	$KI2_{C1}$	0.77nM
Michaelis constant for *mCry1* transcription due to *PER2_CRY1* inhibition	$KI3_{C1}$	3.59nM
Michaelis constant for *mCry1* transcription due to *PER2_CRY2* inhibition	$KI4_{C1}$	3.44nM
Michaelis constant for *mCry1* transcription due to *REV_ERBα* inhibition	$KI5_{C1}$	2.82nM
Michaelis constant for *mCry2* transcription due to *CLK_BMAL1* activation	$KA1_{C2}$	0.69nM
Michaelis constant for *mCry2* transcription due to *RORc* activation	$KA2_{C2}$	2.96nM
Michaelis constant for *mCry2* transcription due to *PER1_CRY1* inhibition	$KI1_{C2}$	4.63nM
Michaelis constant for *mCry2* transcription due to *PER1_CRY2* inhibition	$KI2_{C2}$	2.95nM
Michaelis constant for *mCry2* transcription due to *PER2_CRY1* inhibition	$KI3_{C2}$	3.57nM
Michaelis constant for *mCry2* transcription due to *PER2_CRY2* inhibition	$KI4_{C2}$	2.75nM

Michaelis constant for *mCry2* transcription due to *REV_ERBα* inhibition	$KI5_{C2}$	3.97nM
Michaelis constant for *mRev_erbα* transcription due to *CLK_BMAL1* activation	$KA1_{Re}$	3.15nM
Michaelis constant for *mRev_erbα* transcription due to *PER1_CRY1* inhibition	$KI1_{Re}$	3.56nM
Michaelis constant for *mRev_erbα* transcription due to *PER1_CRY2* inhibition	$KI2_{Re}$	3.62nM
Michaelis constant for *mRev_erbα* transcription due to *PER2_CRY1* inhibition	$KI3_{Re}$	4.71nM
Michaelis constant for *mRev_erbα* transcription due to *PER2_CRY2* inhibition	$KI4_{Re}$	1.23nM
Michaelis constant for *mClk* transcription due to *RORc* activation	$KA1_{Clk}$	1.59nM
Michaelis constant for *mClk* transcription due to *REV_ERBα* inhibition	$KI1_{Clk}$	0.83nM
Michaelis constant for *mBmal1* transcription due to *RORc* activation	$KA1_{Bmal1}$	2.59nM
Michaelis constant for *mBmal1* transcription due to *REV_ERBα* inhibition	$KI1_{Bmal1}$	2.47nM
Michaelis constant for *mRorc* transcription due to *CLK_BMAL1* activation	$KA1_{Rorc}$	4.30nM
Michaelis constant for *mRorc* transcription due to *RORc* activation	$KA2_{Rorc}$	4.89nM
Michaelis constant for *mRorc* transcription due to *PER1_CRY1* inhibition	$KI1_{Rorc}$	3.49nM
Michaelis constant for *mRorc* transcription due to *PER1_CRY2* inhibition	$KI2_{Rorc}$	2.34nM
Michaelis constant for *mRorc* transcription due to *PER2_CRY1* inhibition	$KI3_{Rorc}$	2.71nM
Michaelis constant for *mRorc* transcription due to *PER2_CRY2* inhibition	$KI4_{Rorc}$	2.09nM
Michaelis constant for *mRorc* transcription due to *REV_ERBα* inhibition	$KI5_{Rorc}$	3.36nM
Degradation rate of *mPer1* mRNA	km_{P1}	2.18/h
Degradation rate of *mPer2* mRNA	km_{P2}	0.20/h
Degradation rate of *mCry1* mRNA	km_{C1}	0.22/h
Degradation rate of *mCry2* mRNA	km_{C2}	0.41/h
Degradation rate of *mRev_erbα* mRNA	km_{Re}	0.60/h
Degradation rate of *mClk* mRNA	km_{Clk}	3.19/h
Degradation rate of *mBmal1* mRNA	km_{Bmal1}	1.42/h
Degradation rate of *mRorc* mRNA	km_{Rorc}	1.50/h

Translation rate for *PER1* protein formation	*t_P1*	3.05/h
Translation rate for *PER2* protein formation	*t_P2*	2.38/h
Translation rate for *CRY1* protein formation	*t_C1*	3.94/h
Translation rate for *CRY2* protein formation	*t_C2*	1.69/h
Translation rate for *REV_ERBα* protein formation	*t_REV_ERB*	1.60/h
Translation rate for *CLK* protein formation	*t_CLK*	3.04/h
Translation rate for *BMAL1* protein formation	*t_Bmal1*	4.00/h
Translation rate for *RORc* protein formation	*t_Rorc*	1.39/h
Degradation rate for *PER1* protein	*kp_P1*	2.58/h
Degradation rate for *PER2* protein	*kp_P2*	0.16/h
Degradation rate for *CRY1* protein	*kp_C1*	3.03/h
Degradation rate for *CRY2* protein	*kp_C2*	1.72/h
Degradation rate for *REV_ERBα* protein	*kp_ REV_ERB*	0.31/h
Degradation rate for *CLK* protein	*kp_CLK*	1.52/h
Degradation rate for *BMAL1* protein	*kp_BMAL1*	2.28/h
Degradation rate for *RORc* protein	*kp_ RORc*	3.33/h
Complex association rate for *PER1_CRY1*	*a_P1_C1*	3.57/nM.hr
Complex association rate for *PER1_CRY2*	*a_P1_C2*	3.12/nM.hr
Complex association rate for *PER2_CRY1*	*a_P2_C1*	3.81/nM.hr
Complex association rate for *PER2_CRY2*	*a_P2_C2*	0.95/nM.hr
Complex association rate for *CLK_BMAL1*	*a_CLK_BMAL1*	1.98/nM.hr
Complex dissociation rate for *PER1_CRY1*	*d_P1_C1*	1.32/h
Complex dissociation rate for *PER1_CRY2*	*d_P1_C2*	1.85/h
Complex dissociation rate for *PER2_CRY1*	*d_P2_C1*	1.37/h
Complex dissociation rate for *PER2_CRY2*	*d_P2_C2*	2.42/h
Complex dissociation rate for *CLK_BMAL1*	*d_CLK_BMAL1*	0.97/h

2 Parameter analysis

To exhibit the generality of the conclusions in this study, we randomly generated 10000 sets of parameters for the CRTC1-SIK1 module. The range of the parameters are listed in Table S4. The maximal value of each parameter is determined by 100 times of the value in Table S2.

Table S4: Ranges for the random parameter sets for the CRTC1-SIK1 module

Parameter symbol	Parameter range	Distribution	Parameters in Figure S2B-C	Parameters in Figure S2D-E
V_{0_x}	[0, 1]	Uniform	0.354	0.928

K_{pc_x}	[0, 40]	Uniform	35.084	3.525
K_{pim_x}	[0, 5]	Uniform	0.768	2.103
K_{pex_x}	[0,1000]	Uniform	705.48	410.642
K_{im_x}	[0, 500]	Uniform	121.22	121.16
K_{ex_x}	[0, 10]	Uniform	8.973	7.113
K_{dp_x}	[0, 10]	Uniform	8.791	7.172
$a1$	[0, 45]	Uniform	0.5133	10.7916
V_{0_y}	[0, 5]	Uniform	2.963	4.1697
K_{dp_y}	[0, 30]	Uniform	18.146	15.908
K_{pim_y}	[0, 515]	Uniform	119.717	472.769
K_{pex_y}	[0, 20]	Uniform	19.785	19.521
K_{im_y}	[0, 1]	Uniform	0.293	0.335
K_{ex_y}	[0, 1000]	Uniform	94.956	958.557
K_{pn_y}	[0, 10]	Uniform	9.896	0.448
K_{dpn_y}	[0, 300]	Uniform	22.071	186.086
V_{0_CC}	[0, 32]	Uniform	1.982	1.367
km_{cc}	[0, 30]	Uniform	29.331	11.929
$V0_{sik}$	[0, 1]	Uniform	0.708	0.258
$V0_{SIK}$	[0, 65]	Uniform	51.499	23.503
km_{sik1}	[0, 30]	Uniform	15.171	0.426
km_{SIK1}	[0, 30]	Uniform	25.628	22.283
$b1$	[0, 2500]	Uniform	1879.112	1666.242
$V1_{sik}$	[0, 200]	Uniform	57.945	147.958
$K1_{SIK1}$	[0, 30]	Uniform	8.307	18.772
$K1_{CC}$	[0, 18]	Uniform	16.029	17.297
tot	[0, 100]	Uniform	54.926	84.47
L	$400 \cdot V_{0\_x}$	Uniform	$400 \cdot V_{0\_x}$	$400 \cdot V_{0\_x}$
a	[0, 100]	Uniform	51.896	18.651

For each parameter set, we evaluated the magnitude of light adaptation by the ratio of the stabilized level of CC to the maximal value upon prolonged light exposure.

$$\text{The magnitude of light adaptation} = \frac{\text{the stabilized level of CC}}{\text{the maximal value of CC}}$$

Therefore, smaller ratio implies stronger light adaptation. The simulation results show that 3728 out of 10000 parameter sets yield ratios smaller than 0.8, which implies the occurrence of light adaptation. Among these parameter sets, if *Sik1* mRNA is knocked down, the CRTC1-SIK1 model exhibits weakened light adaptation for 3149 parameter sets (Figure S1). The ratios of light adaptation are weakened more than 20% for 2882 parameter sets. Therefore, this further confirm the conclusion that, the negative feedback loop of SIK1 is necessary for the generation of sufficient light adaptation.

After that, by excluding the sets those destroy the self-oscillation of circadian

clock from the 2882 parameter sets, we obtained 2287 parameter sets to test the phase shift response to short-term light pulse. As the circadian clock with CRTC1-SIK1 module exhibits stable oscillation, a 30-min light pulse is applied at 6h after the peak of *Per1* mRNA. For most parameter sets, the phases of *Per1* mRNA are delayed. By measuring the phase shifts (the time interval between the first peaks of *Per1* mRNA after the light pulse and without light pulse) in WT and *Sik1* knockdown model, we can observe that the mutant condition exhibits larger phase shifts for 2285 out of 2287 parameter sets (Figure S2A). The phase shift is increased by 20% in *Sik1* knockdown model for 1680 parameter sets. We also found that when the amplitude of the circadian oscillation is small, the phase shifts of WT and *Sik1* knockdown model are much larger. Figure S2B-C exhibits an example for this situation, where the difference of phase shifts between WT and *Sik1* knockdown model is -3.38h. Thus, the conclusion that the light adaptation generated by CRTC1-SIK1 module prevents the excessive phase shift still holds true for most parameter sets.

We also noticed that the phase shift in WT is significantly larger than that in *Sik1* mutant model for two parameter sets. Therefore, we checked the magnitude of light adaptation for these parameter sets and found that both WT and *Sik1* mutant model exhibit significant light adaptation (Figure S2D-E). Thus, some other factor may also contribute to the phase shift

The 2287 random parameter sets also provided a further test of the result that the light adaptation can reduce the possibility of singularity. We choose the light stimulus at 6.1h after the peak of *Per1* mRNA for 7 hours, because it is more likely to generate singularity (Figure 7). The result showed that, 41.8% (957 out of 2287) parameter sets can yield MPDs larger than 5 hours for 100 *Sik1* knockdown cells. By contrast, only 23.2% (531 out of 2287) parameter sets generate MPDs larger than 5 hours in 100 WT cells. The situation is similar for the variances of the phases. For 18.45% (422 out of 2287) parameter sets, the differences of the variances are smaller than -5 (the variance in WT minus that in *Sik1* knockdown model). However, for only 4.63% (106 out of 2287) parameter sets, the differences of the variances are larger than 5 (Figure S4).

The above results of the parameter disturbance imply that the regulatory structure of CRTC1-SIK1 network (negative feedback loop) is the essential reason for the light adaptation and preventing the excessive changes of circadian genes.

3 Initial conditions

Initial conditions for the simulations in this article are listed as follows. This initial condition corresponds to CT0h, which is determined by assuming that *Per1* mRNA peaks at CT8.5h according to previous experimental results.

$CRTC1_{PC}$=0.9678, $CRTC1_{C}$=0.0022, $CRTC1_{N}$=0.2084, $CREB_{PN}$=0.1535,

$CREB_{PC}$=0.0094, $CREB_{C}$=0.9663, CC=0.0341, mSik1=0.0333, SIK1=0.0722, mPer1=0.0816, mPer2=0.1774, mCry1=0.2953, mCry2=0.7389, mRev_erb=0.0126, mClk=1.2456, mBmal1=0.7279, mRorc=0.0180, PER1=0.0717, PER2=6.0710, CRY1=0.4809, CRY2=0.9676, REV-ERB=0.0342, CLK=2.4777, BMAL1=1.2687, RORC=0.0073, PER1_CRY1=0.0766, PER2_CRY1=8.3287, PER1_CRY2=0.1060, PER2_CRY2=2.4359, CLK_BMAL1=6.3980

Initial condition for Figure S2B-C

CRTC1PC=0.9099, CRTC1C=0.007952, CRTC1N=5.3226, CREBPN=5.1757, CREBPC=0.03293, CREBC= 0.8664, CC=1.8622, mSik1=0.04738, SIK1=0.0952, mPer1=0.1006, mPer2=0.3599, mCry1=0.112, mCry2=1.842, mRev_erb=0.00717, mClk=1.246, mBmal1=0.728, mRorc=0.1592, PER1=0.1124, PER2=6.344, CRY1=0.2086, CRY2=1.7272, REV-ERB=0.0309, CLK=2.493, BMAL1=1.279, RORC=0.0642, PER1_CRY1=0.0641, PER2_CRY1=3.8118, PER1_CRY2= 0.3191, PER2_CRY2=4.265, CLK_BMAL1=6.5089

Initial condition for Figure S2D-E

CRTC1PC=4.031, CRTC1C=0.04993, CRTC1N=6.5203, CREBPN=1.7366, CREBPC=0.0404, CREBC=4.3107, CC=1.2971, mSik1=0.6166, SIK1=0.6504, mPer1=0.2073, mPer2=0.3268, mCry1= 0.0536, mCry2=1.537, mRev_erb=0.0321, mClk=1.2103, mBmal1=0.7175, mRorc=0.1392, PER1=0.2196, PER2=6.032, CRY1=0.2289, CRY2=1.239, REV-ERB=0.1436, CLK=2.4464, BMAL1=1.2714, RORC=0.05378, PER1_CRY1=0.14035, PER2_CRY1=4.1702, PER1_CRY2=0.4243, PER2_CRY2=2.8223, CLK_BMAL1=6.3786

3 Reference

Jagannath, A., Butler, R., Godinho, S.I.H., Couch, Y., Brown, L.A., Vasudevan, S.R., Flanagan, K.C., Anthony, D., Churchill, G.C., Wood, M.J.A., Steiner, G., Ebeling, M., Hossbach, M., Wettstein, J.G., Duffield, G.E., Gatti, S., Hankins, M.W., Foster, R.G., and Peirson, S.N. (2013). The CRTC1-SIK1 pathway regulates entrainment of the circadian clock. *Cell* 154**,** 1100-1111.

Mirsky, H.P., Liu, A.C., Welsh, D.K., Kay, S.A., and Doyle, F.J., 3rd (2009). A model of the cell-autonomous

mammalian circadian clock. *Proc Natl Acad Sci U S A* 106, 11107-11112.

4 Figure Legend

Figure S1. The differences of the magnitude of light adaptation between WT and *Sik1* knockdown model for 3728 parameter sets. 10000 parameter sets are randomly generated for the CRTC1-SIK1 module. 3728 out of 10000 parameter sets yield the magnitude of light adaptation smaller than 0.8, which implies the occurrence of light adaptation. For each parameter set, we used a vertical bar to represent the difference of the magnitude of light adaptation between WT and *Sik1* knockdown model (the magnitude of light adaptation in WT minus that in *Sik1* knockdown model). It's obvious that the light adaptation is weakened in mutant model for most parameter sets. The maximal synthesis rate of *Sik1* mRNA dependent on CREB/CRTC1 complex $V1_{sik1}$ is decreased by 90% to mimic the knockdown of *Sik1*.

Figure S2. The differences of the phase shifts between WT and *Sik1* knockdown model for 2287 parameter sets. 2287 parameter sets are used to test the phase shift response to short-term light pulse. A 30-min light pulse is applied at 6h after the peak of *Per1* mRNA, when the circadian clock exhibits stable oscillation. **(A)** Each bar represents the difference of the phase shift between WT and *Sik1* knockdown model (the phase shift in WT minus that in *Sik1* knockdown model). For 2285 out of 2287 parameter sets, the mutant model exhibits larger phase shift. **(B-C)** The time series of *Per1* mRNA for the case that the amplitude of the circadian oscillation is small. In this situation, the phase response to light stimulus is further magnified and the difference of phase shift between WT **(B)** and *Sik1* knockdown model **(C)** is -3.38h. **(D-E)** The time series of *Per1* mRNA for the case that the phase shift in WT **(D)** is significantly larger than that in *Sik1* mutant model **(E)**. The parameter sets for **(B-E)** are listed in Table S4, the initial conditions are listed in the section of Initial conditions.

Figure S3. Light adaptation limits the rate of re-entrainment upon the advanced and delayed light/dark cycle. The circadian clock is entrained by 12:12 light/dark cycle for 10 days. Then the dark phase is advanced or delayed from the 11th cycle. The time series of *Per1* mRNA in WT **(A-C)** and *Sik1* knockdown mice **(D-F)** are exhibited. In **(A)** and **(D),** the dark phase is advanced by 12h. In **(B)** and **(E),** the dark phase is delayed by 6h. In **(C)** and **(F),** the dark phase is delayed by 12h. The back curve and the red curve represent the time evolution of *Per1* mRNA without the varied light/dark cycle and after the varied light/dark cycle. The black and white bars on the bottom of horizontal axis represent dark and light phases, respectively.

Figure S4. The differences of the variances of the phases between WT and *Sik1* knockdown model for 2287 parameter sets. The 2287 random parameter sets in Figure S2A are used to test the the result that the light adaptation can reduce the risk of singularity. The light stimulus is applied at 6.1h after the peak of *Per1* mRNA for 7 hours. For each parameter set, we calculated the variances of the phases in 100 slightly

different cells (in 24h after the stimulus). For 18.45% (422 out of 2287) parameter sets, the differences of the variances are smaller than -5 (the variance in WT minus that in *Sik1* knockdown model). However, for only 4.63% (106 out of 2287) parameter sets, the differences of the variances are larger than 5.
